# Fragile X Related Protein 1 Clusters with Ribosomes and Messenger RNAs at a Subset of Dendritic Spines in the Mouse Hippocampus

**DOI:** 10.1371/journal.pone.0026120

**Published:** 2011-10-11

**Authors:** Denise Cook, Maria del Rayo Sanchez-Carbente, Claude Lachance, Danuta Radzioch, Sandra Tremblay, Edouard W. Khandjian, Luc DesGroseillers, Keith K. Murai

**Affiliations:** 1 Department of Neurology and Neurosurgery, Centre for Research in Neuroscience, The Research Institute of the McGill University Health Centre, Montreal General Hospital, Montreal, Quebec, Canada; 2 Département de Biochimie, Université de Montréal, Montréal, Québec, Canada; 3 Division of Experimental Medicine, Department of Medicine, McGill University, Montreal, Quebec, Canada; 4 Neurobiologie Cellulaire, Centre de Recherche Robert Giffard, Université Laval, Québec, Québec, Canada; Inserm U901, France

## Abstract

The formation and storage of memories in neuronal networks relies on new protein synthesis, which can occur locally at synapses using translational machinery present in dendrites and at spines. These new proteins support long-lasting changes in synapse strength and size in response to high levels of synaptic activity. To ensure that proteins are made at the appropriate time and location to enable these synaptic changes, messenger RNA (mRNA) translation is tightly controlled by dendritic RNA-binding proteins. Fragile X Related Protein 1 (FXR1P) is an RNA-binding protein with high homology to Fragile X Mental Retardation Protein (FMRP) and is known to repress and activate mRNA translation in non-neuronal cells. However, unlike FMRP, very little is known about the role of FXR1P in the central nervous system. To understand if FXR1P is positioned to regulate local mRNA translation in dendrites and at synapses, we investigated the expression and targeting of FXR1P in developing hippocampal neurons *in vivo* and *in vitro*. We found that FXR1P was highly expressed during hippocampal development and co-localized with ribosomes and mRNAs in the dendrite and at a subset of spines in mouse hippocampal neurons. Our data indicate that FXR1P is properly positioned to control local protein synthesis in the dendrite and at synapses in the central nervous system.

## Introduction

New protein synthesis is required for long-lasting changes to synapses, changes thought to underlie long-term memory formation [1]. With the discovery of ribosomes and mRNAs in dendrites and at dendritic spines as well as evidence that dendrites can synthesize proteins in the absence of the cell body, we now know that new protein synthesis can occur locally in the dendrite and at spines [2], [3], [4], [5]. Local protein synthesis is thought to support rapid, signal-dependent increases in protein expression required for synaptic plasticity as well as long-term memory formation [6]. Indeed, analysis of single spines using focal uncaging of glutamate has revealed the importance of dendritic protein synthesis in controlling long-lasting structural and physiological changes at individual synapses [7]. Despite the known importance of local protein synthesis in supporting synaptic plasticity, the actual proteins involved in repressing or enhancing mRNA translation at synapses remain poorly defined. A collection of RNA binding proteins has been identified biochemically as components of ribosomes and/or mRNA-containing granules in neurons [8], [9]. However, it remains unclear which RNA proteins are important for regulating local protein synthesis in the dendrite and at dendritic spines [10].

Kanai et al. identified Fragile X Related Protein 1 (FXR1P) as a component of their biochemically isolated neuronal mRNA granule [9]. FXR1P is a member of a small family of RNA binding proteins that also includes Fragile X Related Protein 2 (FXR2P) and Fragile X Mental Retardation Protein (FMRP) [11], [12]. It is well established that loss of FMRP is the cause of Fragile X Syndrome, a syndrome characterized by mental retardation and autism [13], [14], [15]. FMRP controls the trafficking and translation of a subset of mRNAs important for certain forms of protein synthesis-dependent plasticity including mGluR-mediated long-term depression [16], [17], [18]. Interestingly, FXR2P is believed to participate with FMRP in regulating synaptic plasticity and behavior [19], [20], [21]. However, the role of FXR1P in the central nervous system remains unknown. Like FMRP, FXR1P associates with mRNAs and ribosomes in messenger ribonucleoprotein particles (mRNPs) [22], [23], [24], is expressed by neurons [23], [25] and can form homo- and hetero-multimers with FMRP and FXR2P both *in vitro* and *in vivo* [12], [24], [26], [27]. Interestingly, FXR1P can either repress or activate the translation of target mRNAs in non-neuronal cells depending on the cellular context [28], [29]. However, whether FXR1P is positioned to control local protein synthesis at or near synapses remains to be demonstrated. If FXR1P is involved in this process, it should be localized with ribosomes and mRNAs in the dendrite and at spines. We investigated this possibility by determining the expression and localization pattern of FXR1P in the developing mouse hippocampus, a system that is critical for learning and memory processes. We performed co-labeling studies using dissociated mouse hippocampal neurons to more precisely determine if FXR1P colocalizes with protein translational machinery and mRNAs in the dendrite and at spines. Remarkably, we found that FXR1P was highly co-localized with the translational machinery at a subset of spines. These findings suggest that FXR1P is well-positioned to regulate local protein synthesis at synapses and cooperate with other Fragile-X gene family members to control synaptic plasticity.

## Materials and Methods

### Ethics Statement with regards to animal use

All mice used in this study (both male and female) were from a wild-type C57BL/6 strain bred in our animal facility. All experiments involving mice were approved by the Montreal General Hospital Facility Animal Care Committee (Protocol ID#5758) and followed the guidelines of the Canadian Council on Animal Care.

### cDNA plasmids

Farnesylated monomeric RFP in pcDNA3 was described previously [30]. pcDNA 3.1Hyg(+) eGFP-FXR1P (isoform d) and pcDNA3.1Zeo(+) FXR1P (isoform d) plasmids were characterized in a previous publication [28]. EST clones containing full-length mouse cDNAs for FXR1P (isoform a), FXR2 and FMRP (isoform 1) were obtained from Open Biosystems (Clone IDs: 5041635, 9498022, 30532682 respectively). cDNA inserts were PCR amplified and subcloned into pcDNA3 (Clontech) and pcDNA3.1myc-His(-) (B) (Invitrogen). mCherry (courtesy of Dr. R. Tsien) and eGFP were added in-frame to the N-terminus of the Fragile X proteins. All plasmids were verified by sequencing and matched their respective sequences on GenBank, except for the plasmids pcDNA3.1Hyg(+) eGFP-FXR1P and pcDNA3.1Zeo(+) FXR1P, which started with ATG GCG GAC GTG instead of ATG GCG GAG CTG (discrepancy is underlined; see [28]). This discrepancy leads to an amino acid change of MAEL to MADV, which corresponds to the original reported sequence for human FXR1P (Accession number: AAC50155.1, see [11]). This discrepancy was corrected in the pcDNA3.1-FXR1P-myc-his construct. Expression from all constructs was driven by the CMV promoter.

### Antibodies

For detecting FXR1P, we used a rabbit polyclonal antibody against FXR1P (#ML13) which has been described previously [31]. Other antibodies used included mouse monoclonal antibodies against FMRP (mAb1C3;[32]), FXR1P (mAb3FX;[22]), FXR2P (mAbA42, Abcam), myc (Santa Cruz; 9E10), MAP-2 (Sigma-Aldrich; HM-2)and GAPDH (Abcam; ab9484), human anti-ribosomal P antibodies (Immunovision), a rabbit anti-ribosomal large protein L7 (Cell Signaling), a rabbit monoclonal antibody against S6 (Cell Signaling; 5G10) and a goat polyclonal antibody against TIA-1 (Santa Cruz; sc-1751). The specificity of the anti-ribosomal P antibodies for the large ribosomal subunits P0, P1 and P2 was verified previously by others [24].

### HEK cell culture, transfection and western blotting

Human embryonic kidney cells with the SV-40 T antigen (293-T) were cultured in high glucose Dulbecco’s Modified Essential Medium (DMEM, Invitrogen) containing L-glutamine, 110 mg/L sodium pyruvate, 10% fetal bovine serum and 1% penicillin-streptomycin. One day before transfection, cells were split and plated at a density of 1.2×10^6^ cells per 6 cm dish. Cells were transfected with various Fragile X plasmids using Polyfect (Qiagen) according to the manufacturer’s instructions. Cells were lysed after 48 hours in 400 µl RIPA buffer (1% Triton X-100, 1% sodium deoxycholate, 0.1% SDS, 20 mM Tris pH 8.0, 150 mM NaCl and 1 mM EDTA) containing 1 µg/ml each of leupeptin, aprotinin, pepstatin, 10 mM NaF, 1 mM sodium ortho-vanadate and 1 mM PMSF. Lysates were diluted with 3X sample buffer and equal quantities of each lysate were run on a 10% polyacrylamide gel and transferred to PVDF membranes following standard protocols. Membranes were blocked for 40 minutes with 5% BSA/TBS-0.1% Tween, and incubated overnight at 4°C with either #ML13 (1∶100,000) or anti-myc (1∶2000) in TBS-0.1% Tween. The next day membranes were incubated for 1 hour at room temperature with secondary antibodies conjugated to HRP. Chemiluminescent signal was obtained using Amersham ECL Plus Western Blotting Detection Reagents (GE Healthcare) and captured on X-ray film.

### Hippocampal Lysates and Western Blotting

**Figure 1:**
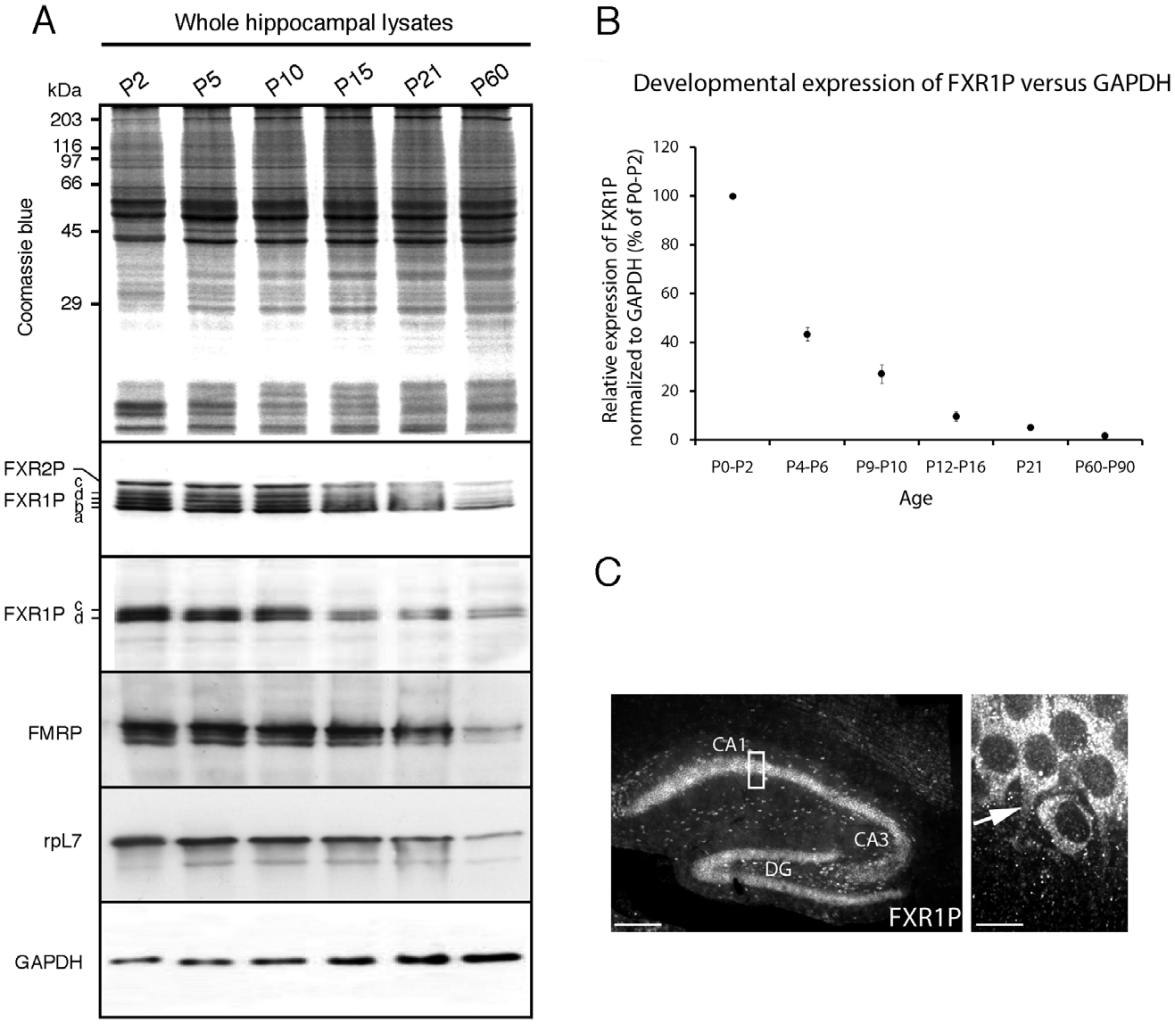
FXR1P is expressed in neurons of the developing hippocampus. **A.** Hippocampal lysates were prepared from mice at different developmental stages (P2 = postnatal day 2) and analyzed for FXR1P, FXR2P, FMRP, L7 ribosomal protein and GAPDH. Isoforms of FXR1P (a.b,c,d), FXR2P, FMRP and ribosomal protein L7 were all highly expressed during early postnatal development in the hippocampus. **B.** FXR1P (isoforms c, d) expression across postnatal development was quantified and normalized against GAPDH expression. FXR1P levels decreased relative to GAPDH. **C.** We immunostained cryostat sections prepared from a P14 mouse with #ML13 and imaged the hippocampus at 10X (left panel). We found that FXR1P was highly expressed in neurons at P14. A 60X image of pyramidal neurons in area CA1 of the hippocampus (box in 10X image) showing FXR1P expression in the cell body and proximal dendrites of CA1 neurons (right panel). Arrow points to a proximal dendrite found in the plane of the image. Scale bar = 60 µm (low magnification) and 10 µm (high magnification)

We dissected out the hippocampus from mice at different points in development (postnatal day 2, 5, 10, 15, 21, 60). Whole cell lysates were obtained by homogenizing the hippocampi in an appropriate volume of RIPA buffer (1% Triton X-100, 1% sodium deoxycholate, 0.1% SDS, 20 mM Tris pH 8.0, 150 mM NaCl and 1 mM EDTA) using a dounce homogenizer. Lysates were left on ice for 30 minutes, sonicated for 10 seconds and spun at 13,200 rpm for 10 minutes. Supernatants were collected and protein concentration was determined using a BCA assay (Pierce). 20 µg of total protein at each time point was run on 10% SDS-PAGE gels and subjected to Coomassie blue staining and immunoblotting as described above. Membranes were incubated with either mAb3FX (1∶2000), mAb1C3 (1∶1), #ML13 (1∶100,000), #anti-L7 (1∶2000) or anti-GAPDH (1∶10,000) as a loading control. We quantified the developmental expression profile of FXR1P relative to GAPDH using densitometry and the ImageJ Gel Analysis Plugin (http://rsb.info.nih.gov/ij/docs/menus/analyze.html#gels). We first normalized the intensity of FXR1P bands to GAPDH by dividing the area measurements returned by ImageJ and then expressed the level of FXR1P as a percentage of the level at the earliest time-point studied (P0-P2). This was repeated across 3 independent experiments. The averages and standard errors of the mean at each developmental time-point are displayed in Figure 1B.

### Cryostat sections and immunohistochemistry

A P14 mouse was transcardially perfused with ice cold Dulbecco’s phosphate buffered saline followed by 20 ml of fixative (4% paraformaldehyde/ 0.1 M phosphate buffer; pH 7.4) using a syringe-pump (Harvard Apparatus). The brain was post-fixed overnight in 10 mL of fixative and transferred to a solution of 30% sucrose/0.1 M phosphate buffer pH 7.4 for 24–48 hours. The brain was then embedded in O.C.T. Compound (EM Sciences) and cut into 30 µm free-floating sagittal sections using a cryostat. Sections were collected in Tris buffered saline (TBS), blocked and permeabilized using 10% normal goat serum/TBS/0.2% Triton-X 100 for 1 hour at room temperature and incubated overnight at 4°C with primary antibody (#ML13; 1∶500) diluted in 1% normal goat serum/TBS/0.2% Triton-X 100. Sections were washed three times for 20 minutes in TBS and incubated with Alexa Fluor goat anti-rabbit 647 (Invitrogen; 1∶500) for 2 hours. Sections were washed three times for 20 minutes and then mounted using SlowFade Gold antifade reagent (Invitrogen). Sections were imaged at 10X (0.4 numerical aperture) using an Ultraview spinning disk confocal system (PerkinElmer, Wellesley, MA) connected to an Eclipse TE2000 (Nikon, Tokyo, Japan) and a cooled CCD 12-bit Hamamatsu ORCA-ER camera. Exposure time was 3000 milliseconds. We created an image of the entire hippocampus by stitching together neighboring single plane images with at least 20% overlap using the Photomerge application of Photoshop CS3 Extended.

### Polyribosome preparation and analyses

Total brain polyribosomes were prepared from 10 day old C57BL/6 mice as described [33] and treated with 25 mM EDTA or 100 µg/ml of RNAse. Ten to fifteen OD at 260 nm were loaded onto 10 ml of 15–45% (w/w) linear sucrose gradients and centrifuged in a Beckman SW40 rotor for 2 hours at 34,000 rpm and 4°C. Gradients were fractionated by upward displacement using an ISCO UA-5 flow-through spectrophotometer set at 254 nm and connected to a gradient collector. Each collected fraction was precipitated overnight at −20°C after addition of 2 volumes of ethanol. The precipitated material was collected by centrifugation at 12,000 rpm for 20 min and solubilized in SDS-sample buffer before immunoblot analyses. FXR1P was detected with mAb3FX, and the ribosomal L7 protein with rabbit anti-L7 serum.

### Dissociated mouse hippocampal neurons

Primary mouse hippocampal neurons were cultured using a modified version of the Banker method [34]. Briefly, astrocytes were isolated from the hippocampi of P1-P2 mice and maintained in Glial Growth Medium (Minimal Essential Medium containing Earle’s salts and L-glutamine supplemented with 10% Horse serum, 0.6% glucose and 1% penicillin/streptomycin (Invitrogen)) until they reached confluency (approximately 7–10 days). At this point, astrocytes were seeded at a density of 80,000 cells/well in 12 well dishes (with 3 paraffin dots/well) coated overnight with poly-D-lysine (0.1 mg/ml). After 4 days medium was changed to Neuronal Growth Medium (Neurobasal A containing 2% B27, 1 mM Glutamax and 1% penicillin/streptomycin (Invitrogen)) to condition the medium overnight. The next day, hippocampi from P0 mice were dissociated in Neuronal Growth Medium containing 1 mg/ml papain and 0.02% BSA for 15 minutes at 37°C. Hippocampi were then transferred to Neuronal Growth Medium containing 1% trypsin inhibitor (Sigma) and 1% BSA and triturated using a fire-polished pipette. Cells were then resuspended in Neuronal Growth Medium and counted. We plated neurons at a density of 80,000 cells/well onto poly-L-lysine (0.1 mg/ml) coated coverslips (15 mm, Fisher). After 3 hours, coverslips with neurons were transferred onto the paraffin dots and placed face-up on the astrocyte feeder layer. After 3 days, 3 µM Ara-C was added to inhibit glial growth. One-third of the medium was changed every 3–4 days.

### Lipofectamine 2000 transfection of primary hippocampal neurons

Primary neurons were transfected at 7 or 14 days *in vitro* using Lipofectamine 2000. Briefly, 1.5 µg of cDNA and 3 µl Lipofectamine 2000 (Invitrogen) were separately diluted in 100 µl of Minimum Essential Medium and incubated for 5 minutes at room temperature. DNA/Lipofectamine complexes were combined, vortexed for 2 seconds and incubated for 30 minutes. During this time, coverslips with neurons were transferred to wells in a separate 12 well dish containing 1 ml of pre-warmed plain Neurobasal A Medium. The DNA/Lipofectamine complexes (200 µl) were then added dropwise to each well. After 3–4 hours the coverslips were returned to the astrocyte feeder layer. We routinely checked the health of our transfected neurons using MAP2 labelling [35]. We found that unhealthy transfected neurons had little or no MAP2 staining. We obtained approximately 5–15 healthy transfected cells per coverslip using this method.

### Immunostaining of dissociated neurons

Neurons were fixed at 7 or 14 days *in vitro* using ice cold 4% paraformaldehyde/4% sucrose/0.1 M phosphate buffer for 15 minutes. Neurons were then washed once with a solution of Dulbecco’s phosphate buffered saline (DPBS)/10 mM glycine and permeabilized using a solution of DPBS/10 mM glycine/0.2% Triton-X 100 for 15 minutes at room temperature. We then washed the neurons using DPBS/10 mM glycine/0.1% Triton-X 100 and blocked them in 5% BSA/DPBS for 1 hour at room temperature. Neurons were then incubated overnight at 4°C with primary antibodies diluted in 5% BSA/DPBS (mouse anti-MAP2 HM-2 (Sigma-Aldrich, 1∶200); #ML13, 1∶200; P0, 1∶500; rabbit anti-S6, 1∶200, goat anti-TIA-1 (Santa Cruz, 1∶200), FMRP mAb1C3 (tissue culture supernatant, neat), FXR2P mAbA42 (Millipore, 1∶50), mouse anti-AGO2 (Abnova, 1∶300), mouse anti-PAK1 (Abnova, 1∶300)). Neurons were washed 3 times for 5 minutes using DPBS/0.1% Triton-X 100 and incubated with suitable Alexa Fluor conjugated secondary antibodies (−488, −568, −647) diluted to 1∶300 in 5% BSA/DPBS. Neurons were then washed three times and mounted using SlowFade Gold Reagent (Invitrogen).

### Fluorescence in situ hybridization of dissociated neurons

Oligonucleotide probes (27-mer poly(dT) or poly(dA)) were 3' end labelled with digoxigenin (DIG) as indicated by the manufacturer (Roche). DIG incorporation was checked by dot blot. Fixed cells were subjected to fluorescence *in situ* hybridization (FISH) with the DIG-labelled poly(dT) or control poly(dA) probes as described previously with some modifications [36]. Cells were washed in PBS containing 5 mM MgCl_2_ (PBSM) and 0.1 M glycine, dehydrated in 50% ethanol and finally in 70% ethanol for at least 3 hours. Cells were then rehydrated with PBSM, permeabilized with 0.25% Triton-X 100 in PBSM and washed with PBSM. The cells were treated for 10 minutes with acetic anhydride in 0.1 M TEA, washed with 1X SSC and equilibrated with 1X SSC and 20% formamide for 5 minutes at room temperature. Probe mixture (10 ng) was dried down with *Escherichia coli* tRNA (10 mg) and sonicated salmon sperm DNA (10 mg), then suspended in 15 µl of 40% formamide and 4X SSC pH 7.0. Probes were mixed with 15 µl of hybridization buffer (20% dextran sulfate, 0.4% BSA and 4 mM vanadyl ribonucleotide complex). The coverslips were covered with parafilm containing 30 µl of probe mixture and hybridized overnight at 37°C in a humid chamber. After hybridization, coverslips were washed for 20 minutes in 20% formamide/1X SSC at 37°C and followed by three 10 minute washes in 1X SSC and two 20 minute washes in 0.1X SSC at room temperature. Hybridized probes, eGFP-FXR1P fusion protein and endogenous FXR1P and P0 were detected by immunofluorescence using an anti-DIG antibody conjugate with rhodamine (1∶25; Roche), a mouse anti-GFP antibody (1∶250; Roche Molecular Biochemicals), a rabbit anti-FXR1P antibody (1∶100; #ML13) and a human anti-P0 antibody (1∶200, Immunovision) respectively. The primary antibodies were incubated overnight at 4°C. Secondary antibodies used were Alexa 488-conjugated goat anti-mouse IgG (1∶500; Molecular Probes) and Alexa 647-conjugated goat anti-human IgG (1∶500; Molecular Probes).

### Imaging of dissociated neurons

Neurons were imaged at 60X using an oil immersion objective (60X Plan Fluor 1.25 numerical aperture) using an Ultraview spinning disk confocal system (PerkinElmer, Wellesley, MA) connected to an Eclipse TE2000 (Nikon, Tokyo, Japan). Excitation band pass filters are as follows: 488, 568 and 647 nm (+/−10 nm). Emission band pass filters are as follows: 525+/−50 nm, 607+/− 45 nm and 700+/−75 nm. Exposure time was adjusted to obtain maximal signal to noise without saturating pixels in the dendrites (as a consequence, pixels within the cell body were sometimes saturated; however, the cell body was never used for analysis). Single plane images or image stacks were acquired using a Z-step of 0.6 µm.

### Colocalization Analysis

We used 0.1 µm, 0.5 µm and 4 µm Tetraspeck Fluorescent Microspheres (Invitrogen) to check for chromatic aberration. These microspheres emit fluorescence in the blue/green/red/far red channels and we used them to test whether the green/red/far red signals properly overlap. We found close apposition of signals in the green and red channels with a slight off-set in the far red channel. Therefore, most of our colocalization experiments were performed using red and green signals. Control experiments using single primary and secondary antibodies, secondary antibodies only and single primary antibodies with both secondary antibodies were performed to rule out bleed-through of signals and cross-reactivity of antibodies. We quantified colocalization between FXR1P and ribosomal proteins using the Intensity Correlation Analysis Plugin in ImageJ [37]. We first converted 16-bit monochromatic single plane images or maximum projection images to 8-bit, selected a background region of interest (ROI) and subtracted background using the ImageJ plugin “Background subtract from ROI” with default setting of 2 standard deviations (except for *in situ* hybridization experiments where 0.5 standard deviations was used). No thresholds were set. We then drew a line ROI along a dendrite and ran the Intensity Correlation Analysis (ICA) Plugin. At least 2 dendrites per cell were analyzed. This plugin generates multiple coefficients of colocalization, including Pearson’s (Rr), Mander’s M1 and M2 and the Intensity Correlation Quotient (ICQ). Since each of these values is influenced in different ways by image quality, background and differences in signal intensity, relying on any one measure can misrepresent the degree of colocalization in images [38]. Thus, for a more complete understanding of the degree of colocalization, we have decided to present the results obtained from each of these measures. Pearson’s coefficient measures how correlated the intensities of both channels are and varies between −1 and 1. A value from 0.5 to 1.0 indicates colocalization [38]. M1 and M2 describe how much of the green signal overlaps with red signal and vice-versa. They vary between 0 and 1 with anything more than 0.5 indicating colocalization. The ICQ measures whether the signals in both channels vary in synchrony. It is calculated on a pixel by pixel basis by first subtracting the mean intensity from the pixel intensity of each channel and then multiplying the values obtained for both channels. If the signals vary in synchrony, then the differences from the mean will be both positive or both negative, resulting in a positive multiplication product. The ICQ is then calculated by summing up the number of pixels with positive multiplication products (product of the differences from the mean (PDM)), dividing by the total number of pixels and subtracting 0.5. This results in a value that varies between −0.5 (segregated staining) and 0.5 (perfect colocalization). A value close to 0 signifies random staining. To calculate the number of overexpressed FXR1P clusters containing ribosomal markers we ran the ICA Plugin and generated an image displaying the location of the positive PDMs. This image contains PDMs from pixels that are both above the mean (+×+) and below the mean (−×−). Since pixels below the mean are mostly 0, 0 pixels, we used the PDM image for pixels above the mean. This image was thresholded, converted to a binary image and the number of particles was calculated using the Analyze Particles plugin in ImageJ. This process was repeated for the FXR1P image. The number of FXR1P clusters containing colocalized signal was determined by dividing the number of colocalized particles by the total number of FXR1P clusters.

### Mouse organotypic hippocampal slices

Hippocampal slices were prepared according to previously published methods [30], [39]. Briefly, the hippocampus was removed from P7 mouse pups and cut into 300 µm transverse slices using a tissue chopper (McIllwain). Approximately 4–6 slices were placed in a circle in the center of a semi-porous tissue culture insert (0.4 µm pore size; Millipore) and maintained in culture media consisting of 50% Minimum Essential Medium (+ Glutamax), 25% heat-inactivated horse serum, 25% Hank’s Balanced Salt Solution and 6.5 mg/ml D-glucose (Sigma). Medium was replaced every two days.

### Gene Gun transfection and imaging of CA1 pyramidal cells

We prepared the cartridges for transfection according to previously published methods [40]. Briefly, we precipitated 25 µg of eGFP-FXR1P and 25 µg of RFPf plasmid DNA onto 25 mg of 1.6 µm gold particles (Bio-Rad) using 100 µl 0.05 M spermidine and 100 µl 1 M CaCl_2_. Gold particles with precipitated DNA were then washed three times with 1 ml absolute ethanol, resuspended in 3 ml of 0.05 mg/ml polyvinylpyrrolidone in absolute ethanol (PVP, Bio-Rad) and drawn into pre-dried Tefzel tubing. The tubing was placed into the Bio-Rad preparation station and the gold particles were allowed to settle for 3 minutes. We then slowly withdrew the ethanol and allowed the tubing to dry for 5 minutes. Hippocampal slices were transfected at 7 days in vitro using helium at 110–130 psi. A 3.0 µm membrane filter (Millipore) was placed between the gene gun nozzle and the hippocampal slices to decrease the shock-wave and improve transfection efficiency. Slices were fixed 48 hours after transfection and imaged using the 60X oil immersion objective and confocal microscopy as described previously. The primary apical dendrites of CA1 pyramidal cells (∼100 µm from the cell body) in both green (eGFP-FXR1P) and red (RFPf) channels were acquired using Metamorph (Molecular Devices). Z-stacks were produced using a z-step of 0.3 µm. We imaged 17 CA1 apical dendrites across multiple slices cultured from four mouse litters.

### Analysis of FXR1P cluster location in CA1 dendrites

To analyze FXR1P cluster location, we first created separate maximum projection images for eGFP-FXR1P and RFPf. The RFPf images were thresholded linearly in Photoshop (Adobe Systems, Seattle, WA) and imported into Reconstruct. For each image, using only the RFPf channel, (and therefore blind to the location of FXR1P clusters) we measured the length of a small dendritic segment (30–70 µm) and counted the number of spines along that length (30–80 spines). We then manually traced the total perimeter and spine head perimeter of each of the spines along the segment. The perimeter drawings were saved and overlaid with the eGFP-FXR1P images. We counted the number of FXR1P clusters along the dendritic segment. A cluster was defined as being at a spine if it was found within the spine’s traced perimeter. A cluster was scored as being in the spine head if it was found within the spine head perimeter and as being in the base/neck if it was found outside the spine head perimeter. For spines lacking clear spine heads (ie. stubby spines), the cluster was scored as being in both the spine head/base/neck (“all”).

### Statistical Analysis

All statistical analyses were performed using R (http://www.R-project.org) [41]. The package Hmisc was used to calculate means and standard deviations [42]. All graphs were produced in R using ggplot2 [43]. Confidence intervals were calculated using resampling techniques (bootstrapping) implemented in the base R package boot using values from individual observations (cells). Standard errors for colocalized granules were calculated using the average percent colocalization from each independent culture.

## Results

### FXR1P is expressed in neurons of the developing hippocampus

In contrast to FMRP, very little is known about the expression and localization pattern of FXR1P in the developing and adult mouse brain. In order to determine whether FXR1P is in a position to regulate local protein synthesis in neurons we first examined the expression of FXR1P in the developing mouse hippocampus. We were particularly interested in the expression of FXR1P in the first three postnatal weeks since this corresponds to a time period when there is the highest presence of translational machinery in dendrites and at spines and maximal synapse growth [44]. Whole lysates were prepared from mouse hippocampi at different developmental stages, loaded onto an SDS-PAGE gel and analyzed by Coomassie blue staining. Staining revealed even loading of total protein with only subtle changes in the intensity of labeled bands during development (Figure 1A). To determine whether FXR1P expression changes during development, we used mAb3FX which detects all FXR1P isoforms (a to f). The results showed that FXR1P isoforms a, b, c, and d were highly expressed in early postnatal development (P2–P10) with a substantial drop in expression after P15 (Figure 1A). Since mAb3FX also reacts with FXR2P, we further resolved the expression of the 78 kDa (iso d) and 80 kDa (iso c) isoforms of FXR1P using the FXR1P specific antibody #ML13 (Figure 1A, Figure S1A), which gave a similar pattern as mAb3FX. As expected, the muscle-specific long isoforms (e, f), which run at 84–88 kDa [22], were not present in hippocampal lysate. We also blotted for FMRP and observed a similar decrease in expression across development. Importantly, we observed that the decay of the ribosomal protein L7 was similar to the Fragile X proteins. This suggests a global decrease in the abundance of translational machinery as compared to other proteins such as GAPDH (Figure 1A). Normalizing FXR1P levels with GAPDH expression showed a significant decrease in FXR1P expression across postnatal development compared to GAPDH (Figure 1B). These results indicate that FXR1P was highly expressed during early postnatal stages, a time when synapses are actively forming and reorganizing during hippocampal development.

To define the cellular localization pattern of FXR1P, we performed immunofluorescence labeling on sections from mouse hippocampus at multiple developmental time points using the FXR1P specific serum #ML13. FXR1P (isoforms c and d) were enriched in the cytoplasm of pyramidal and non-pyramidal neurons at all time points studied (P10, P12, P14, P16, P18, P30 and P63). A representative image from postnatal day 14 is shown in Figure 1C. At high magnification the majority of the FXR1P staining was found in the perinuclear cytoplasm and proximal dendrites of pyramidal neurons and observed as small punctae in the stratum radiatum. FXR1P was also detected in large interneurons in the stratum oriens, radiatum, and lacunosum moleculare. In contrast, we observed very limited expression of FXR1P in glia. Control experiments with application of secondary antibody alone did not reveal significant labeling (Figure S1B). Therefore, FXR1P is strongly expressed by developing neurons in the mouse hippocampus and localized in dendrites.

**Figure 2:**
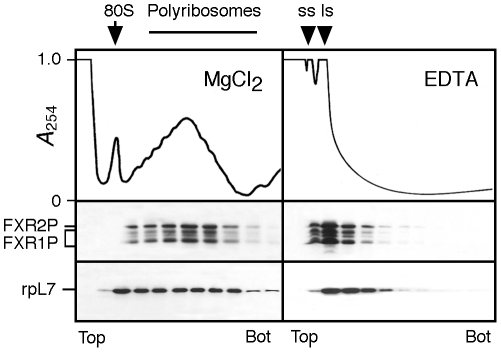
FXR1P is associated with polyribosomes in mouse brain extracts. Aliquots of native polyribosomes and EDTA treated polyribosomes were loaded onto linear 15–45% (w/w) sucrose gradients and centrifuged for 2 hr at 34 000 rpm at 4°C in a Beckman SW40 rotor. Each collected fraction was assayed for the presence of FXR1P and L7 ribosomal protein. Fractions from the top to the bottom of the gradient are shown from left to right and the position of the 80 S ribosome monomer is indicated. SS, LS: ribosomal small and large subunits, respectively.

While it is established that FXR1P, similarly to FMRP, is physically associated with translation machinery in non-neural cells [22], [28], [45], it has been assumed that this is also the case in the central nervous system. To determine whether FXR1P is associated with the translational apparatus in brain, total polyribosomes were prepared from P10 brain as previously described [33] and analyzed by velocity sedimentation through sucrose density gradients. In the presence of Mg^2+^, all FXR1P isoforms were detected in fractions corresponding to heavy sedimenting polyribosomes (Figure 2). The presence of the ribosomal protein L7 in the fractions was used as a control. Upon addition of EDTA, which dissociates ribosomes into their subunits concomitant with the release of free mRNP complexes, FXR1P was displaced to the upper part of the gradient with sedimentation values corresponding to mRNPs. Finally, treatment with RNase A resulted in the complete destruction of polyribosomes and all FXR1P isoforms were displaced to the top fractions of the gradient (data not shown). Since mAb3FX was used in this analysis, these results established that both FXR1P and FXR2P co-sediment in the same fractions (Figure 2).

### FXR1P forms clusters in the dendrite and at spines

**Figure 3:**
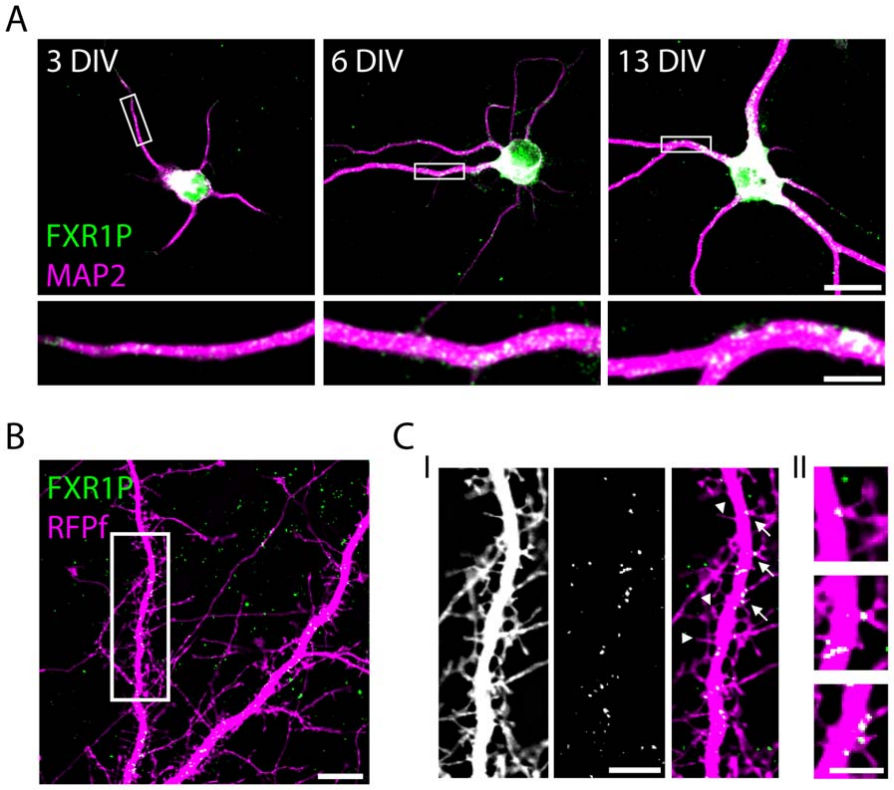
FXR1P forms clusters along the dendrite and at a subset of spine-like protrusions. **A.** We fixed dissociated hippocampal neurons at different developmental time-points and immunostained them with antibodies against FXR1P (#ML13; green) and MAP2 (dendritic marker; magenta). FXR1P formed clusters along dendrites at all developmental time-points. High magnification views of the segments outlined in white are shown below each image. We noted an increase in cluster size and intensity over time. Scale bar = 20 µm (low magnification) and 5 µm (high magnification). **B.** We transfected hippocampal neurons at 14 days *in vitro* with a plasmid encoding membrane targeted red fluorescent protein (RFPf) and immunostained for FXR1P. The single plane FXR1P image was thresholded to highlight the brightest clusters. FXR1P was found in clusters along the dendrite and at a subset of spine-like protrusions. Scale bar = 10 µm**. C.** (**I)** High magnification view of the segment of dendrite boxed in white in B. FXR1P clusters were found in the base, neck or head of a subset of dendritic spine-like protrusions. Arrows denote spine-like protrusions with an FXR1P cluster; arrowheads denote spine-like protrusions without an FXR1P cluster. DIV = days *in vitro*. Scale bar = 5 µm **C. (II)** High magnification view of the FXR1P positive spine-like protrusions labeled in C (I). Scale bar = 2.5 µm (high magnification).

Having established that FXR1P was expressed by developing neurons in the mouse hippocampus and present in dendrites, we performed a more detailed subcellular characterization of endogenous FXR1P along dendrites. To do this, we used low-density dissociated mouse hippocampal neurons which allowed us to resolve the discrete localization of FXR1P in isolated dendrites and to colocalize FXR1P with other proteins. Similar to what we found *in vivo*, FXR1P was highly expressed in the perinuclear region and found as individual punctae in MAP2-positive dendrites (Figure 3A). Large punctae were especially prevalent in proximal dendritic regions while smaller punctae were found in more distal dendritic segments. FXR1P was found in punctae of different sizes that, in general, became progressively larger with the age of neuronal cultures (Figure 3A). Due to the heterogeneous size of these punctae and the fact that FXR1P is known to multimerize, we will refer to these punctae as “clusters”. We next followed up on the distribution of FXR1P clusters with respect to dendritic spines and filopodia, a subset of which are known to contain protein translation machinery at their bases [2]. To fully delineate dendrites, filopodia and spines we used a construct encoding farnesylated red fluorescent protein (farnesylated RFPf) which is targeted to the cell membrane. We transfected hippocampal neurons with an RFPf construct at 14 days *in vitro* and then immunostained for endogenous FXR1P (Figure 3B). Upon close examination of FXR1P clusters we found that some of these clusters were in close proximity with the base of a subset of dendritic filopodia or spine-like extensions (Figure 3C, I and II). FXR1P was also detected in axons, however the clusters were smaller and more infrequent than in the dendrites (data not shown). These experiments demonstrate that FXR1P accumulates in discrete clusters in the dendrite and at dendritic spine-like protrusions.

### FXR1P colocalizes with ribosomal subunits and mRNAs in clusters along the dendrite

**Figure 4:**
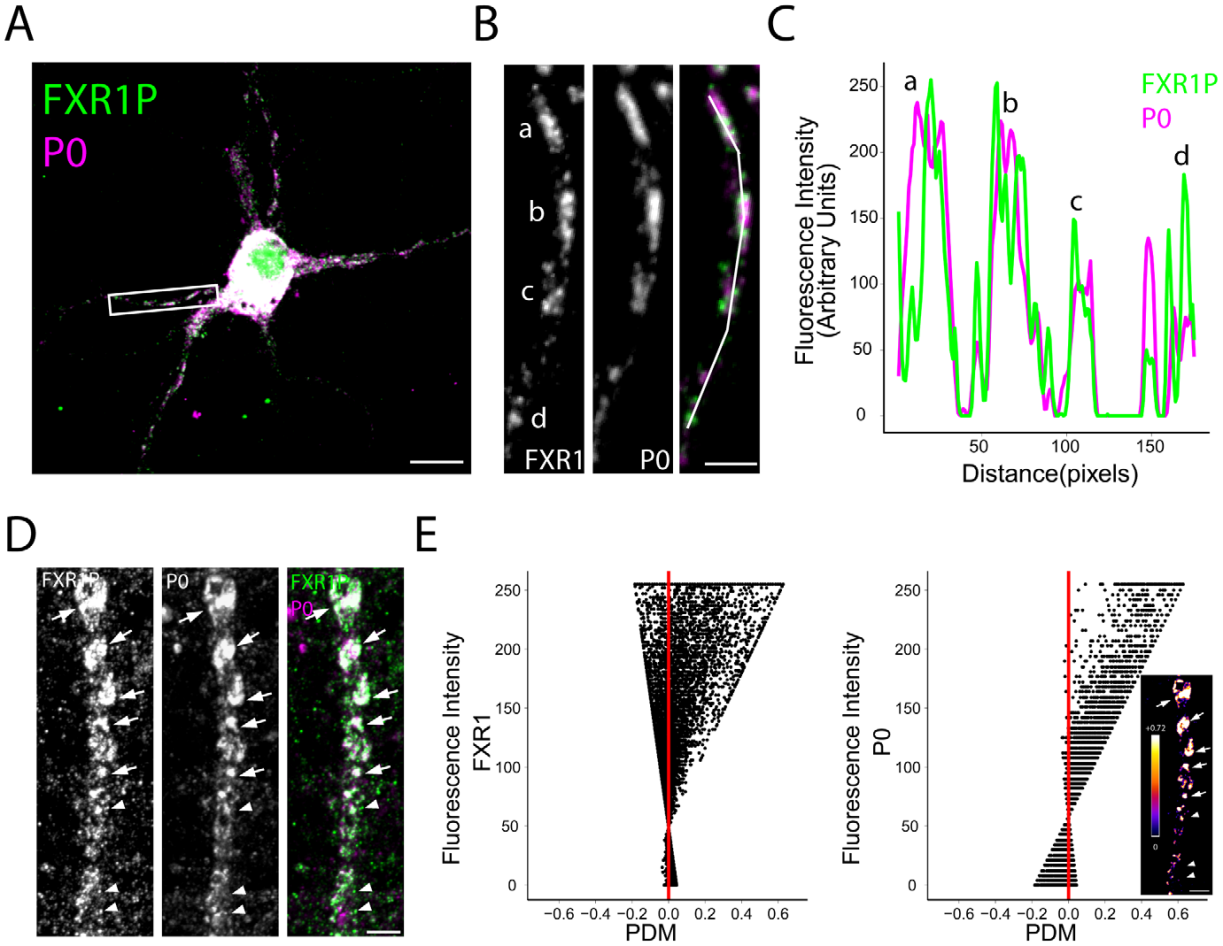
FXR1P colocalizes with ribosomes in clusters along the dendrite. **A.** Immunostaining of dissociated hippocampal neurons at 14 days *in vitro* with anti-FXR1P (#ML13) and anti-P0 shows a high degree of colocalization between FXR1P and P0 (white signal). Scale bar = 10 µm. **B.** High magnification view of the dendritic segment outlined in A showing colocalization between FXR1P and P0 in clusters along the dendrite. Scale bar = 2.5 µm. **C.** Graph demonstrating the covariance in the fluorescence intensities of FXR1P and P0 along the dendritic segment traced in B. **D, E.** Example of the results obtained from the Intensity Correlation Analysis (ICA). Images showing FXR1P, P0 and merged staining (D). Arrows point to colocalized clusters of FXR1P/P0, whereas arrowheads point to bright FXR1P clusters lacking P0. Scale bar = 5 µm. In E, the fluorescence intensity of FXR1P and P0 was plotted against the Products of the Differences from the Mean (PDM) of that pixel. Pixels where fluorescence intensities are correlated are shown to the right of the red line; uncorrelated pixels are shown on the left. These graphs show that a large number of high intensity P0 and FXR1P pixels are correlated. However, a fraction of high intensity FXR1P pixels are not correlated with P0 intensity, whereas a fraction of low intensity P0 pixels are not correlated with FXR1P intensity. (Inset) Image showing the positive PDM produced using the ICA plugin in ImageJ. For clarity, only the PDMs for pixels with intensities above the mean are shown. An intensity lookup table has been applied to the image and is shown on the right. Scale bar = 5 µm.

**Figure 5:**
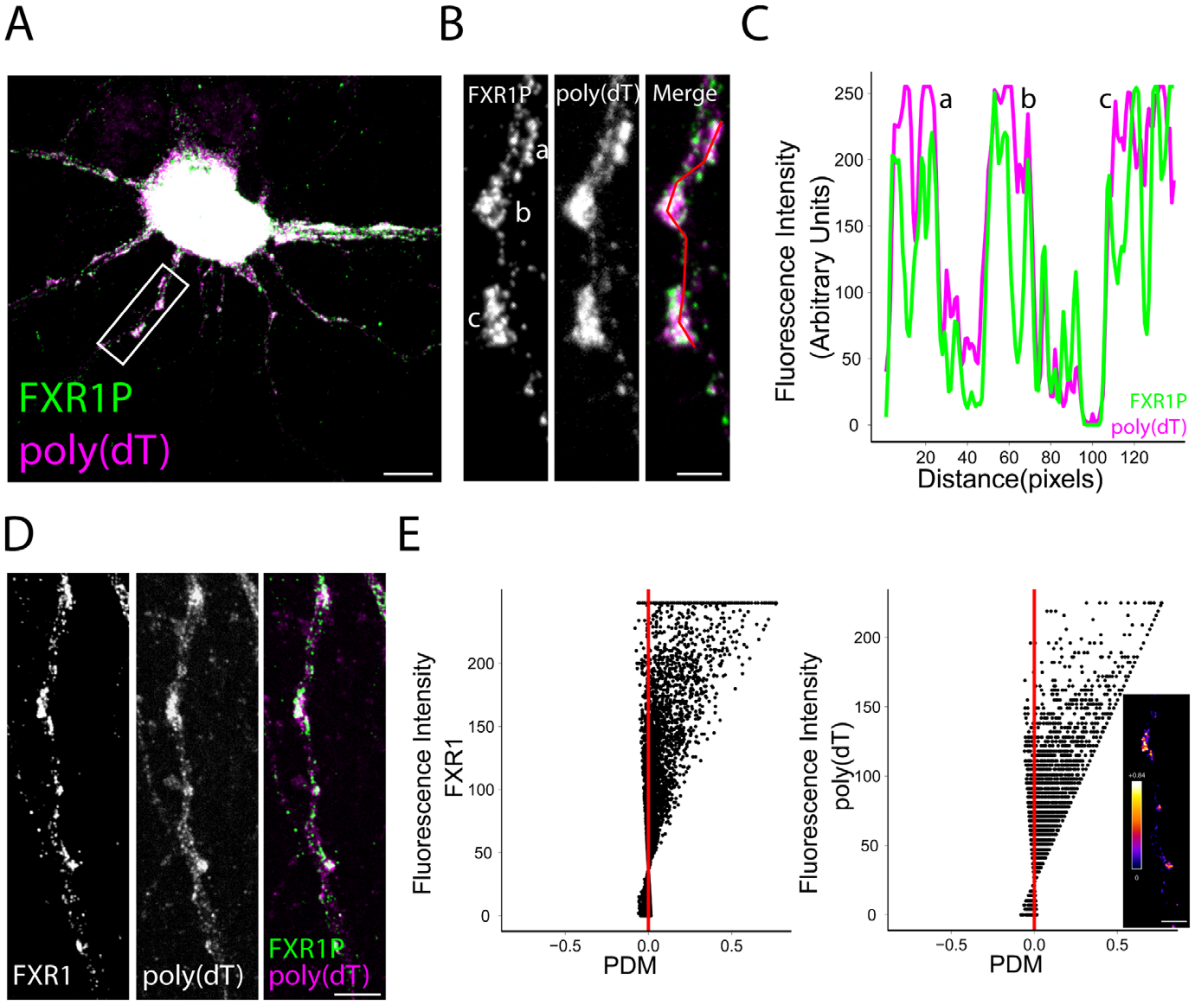
FXR1P colocalizes with mRNAs in clusters along the dendrite. **A.** We performed fluorescence *in situ* hybridization on dissociated hippocampal neurons at 14 days *in vitro* using a digoxigenin-labeled poly(dT) probe to detect polyadenylated mRNAs. *In situ* hybridization was followed by immunostaining using anti-FXR1P (#ML13) and anti-P0 antibodies (data not shown). This merged image shows a high degree of colocalization between FXR1P and poly(dT) (white signal). Scale bar = 10 µm. **B.** High magnification view of the dendritic segment outlined in A showing colocalization between FXR1P and poly(dT) in clusters along the dendrite. Scale bar = 2.5 µm**. C.** Graph showing covariance in the fluorescence intensities of FXR1P and poly(dT). **D, E.** Example of results obtained from the Intensity Correlation Analysis (ICA). D. Images showing FXR1P, poly(dT) and merged staining. Scale bar = 5 µm E. The fluorescence intensity of poly(dT) and FXR1P was plotted against the Product of the Differences from the Mean (PDM) of that pixel. Pixels where fluorescence intensities are correlated are shown to the right of the red line; uncorrelated pixels are shown on the left. These graphs show that the majority of FXR1P and poly(dT) pixels are correlated. Inset. Image showing the positive PDMs produced using the ICA plugin in ImageJ. For clarity, only the PDMs for pixels with intensities above the mean are shown. An intensity lookup table has been applied to the image and is shown to the right. Scale bar = 5 µm.

We have shown that FXR1P physically associates with polyribosomes in the developing mouse brain (Figure 2). However, this analysis does not allow us to determine whether this association takes place in dendrites and at spines. If FXR1P plays a role in local protein synthesis, then it should colocalize in dendrites with components of the translational machinery, for example ribosomes and/or mRNAs. We investigated this by quantifying the degree of colocalization between FXR1P and ribosomes or mRNAs in dissociated hippocampal neurons at 14 days *in vitro* (Figures 4, 5). Immunostaining for the large ribosomal subunit P0 was used to detect ribosomes while fluorescence *in situ* hybridization (FISH) with a poly (dT) probe was used to detect polyadenylated mRNAs. FISH labeling with a poly (dA) probe was used in control experiments (Figure S2). First, using a qualitative method to look at colocalization, we saw a large amount of overlapping signal on the merged image of FXR1P and P0 as well as FXR1P and mRNAs in both the perinuclear region and proximal dendrites (Figures 4A and 5A). We used ImageJ to measure the intensity changes of the two signals along the dendritic segment shown in Figures 4B and 5B. This displayed a strong co-variance in the FXR1P/P0 and FXR1P/mRNA signals (Figures 4C and 5C). However, since determining the degree of overlap with these methods is subjective and influenced by differences in intensities between the two channels, we used the Intensity Correlation Analysis (ICA) Plugin in ImageJ to quantify the degree of colocalization in dendritic segments using multiple methods [37] (see methods). All coefficients indicated significant levels of colocalization between FXR1P/P0 and FXR1P/mRNA (Table 1). Importantly, ICA reveals not only the degree of correlated signal but also non-correlated signal. Typical results obtained from this type of analysis are shown in Figures 4D, E and 5D, E. Figures 4D and 5D present the grayscale and merged images of the dendritic segments used in ICA. Plots of fluorescence intensity versus product difference of the mean (PDM) are shown in Figures 4E and 5E. A positive PDM indicates a pixel with correlated FXR1P and P0 or mRNA intensities (right of the red line), whereas a negative PDM indicates a pixel with non-correlated FXR1P and P0/mRNA intensities (left of the red line). The majority of FXR1P and P0/mRNA pixels fall to the right of the red line, indicating a high level of co-dependence of the signals. The location of these correlated pixels is shown in the inset. Interestingly, a number of high intensity FXR1P pixels contained uncorrelated P0 intensities (left of the red line), whereas most of the pixels for FXR1P and mRNA were correlated (Figures 4E and 5E). This demonstrates that most FXR1P clusters contain mRNAs and ribosomes and a fraction of FXR1P clusters lack ribosomes. These collective results demonstrate that FXR1P clusters are colocalized with protein synthesis machinery in dendrites.

**Table 1:** FXR1P colocalizes with ribosomes and mRNA (Means, 95% Confidence Intervals).

	Pearson’s Coefficient	Mander’s 1^a^	Mander’s 2^b^	Intensity Correlation Quotient
**FXR1P / P0** c	0.63 (0.59−0.67)	0.86 (0.80−0.93)	0.89 (0.87−0.92)	0.26 (0.25−0.28)
**FXR1P / mRNA** d	0.74 (0.70−0.77)	0.97 (0.96−0.99)	0.90 (0.87−0.93)	0.31 (0.28−0.32)
**FXR1P / PSD95** e	−0.05 (N/A)	0.22 (N/A)	0.76 (N/A)	0.11 (N/A)

a Overlap of FXR1P with label of interest.

b Overlap of label of interest with FXR1P.

c Number of independent cultures = 4; Number of cells = 38.

d Number of independent cultures = 2; Number of cells = 21.

e Number of independent cultures = 1; Number of cells = 6.

**Figure 6:**
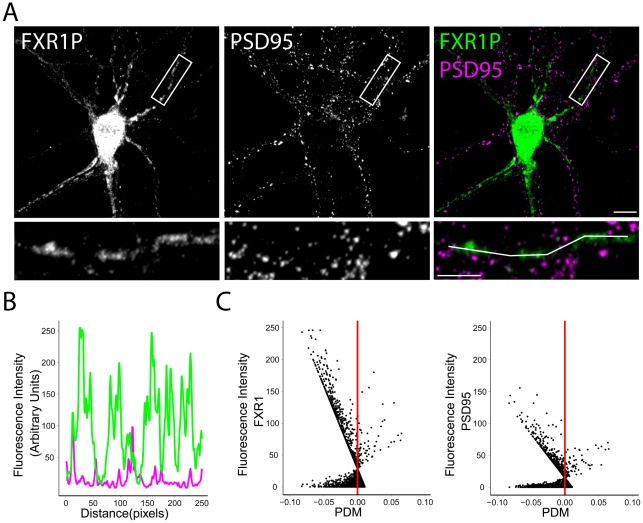
FXR1P does not colocalize with PSD95. **A.** We immunostained dissociated hippocampal neurons at 14 days *in vitro* using anti-FXR1P (#ML13) and anti-PSD95 antibodies. Single channel and merged images show the lack of colocalization between FXR1P and PSD95. Scale bar = 10 µm. High magnification view of the dendritic segment outlined above are shown below. **B.** Graph showing the lack of covariance in the fluorescence intensities of FXR1P and PSD95 along the drawn line shown in A. **C.** Intensity correlation analysis of the segment shown in A. The fluorescence intensity of each PSD95 and FXR1P pixel was plotted against the Product Difference of the Mean (PDM) of that pixel. Pixels where fluorescent intensities are correlated are plotted to the right of the red line; uncorrelated pixels are plotted on the left. These graphs show that most of the pixels lie to the left of the red line, demonstrating a lack of colocalization between FXR1P and PSD95.

To validate our method of quantifying colocalization, we repeated the analysis using co-immunostaining of FXR1P and PSD95. PSD95 is discretely localized to postsynaptic sites including the heads of dendritic spines [46]. As shown in Figure 6A, the staining patterns of FXR1P and PSD95 are different. Measuring the intensities of the two signals along the dendritic segment shown in the bottom panel of Figure 6A confirms the lack of co-variance in the two signals (Figure 6B). In addition, most of the measures of colocalization demonstrated a lack of colocalization between the two channels (Table 1), and the intensity correlation analysis showed that most of the FXR1P and PSD95 pixels had PDM values less than 0, demonstrating a lack of co-dependence of the two signals (Figure 6C). These results demonstrate a lack of colocalization between FXR1P and PSD95 and is consistent with findings showing that protein synthesis machinery is concentrated mostly near the base of dendritic spines and not at the postsynaptic density [44].

Previous studies have found that FXR1P can interact with its homologues FMRP and FXR2P [12], with the miRNA-induced silencing complex (RISC) protein, argonaute 2 [29] and with the actin modulator, PAK1 [47]. To determine whether FXR1P colocalizes with these proteins in neuronal dendrites we performed immunostaining for FXR1P, P0 and each of these interacting proteins. Qualitatively, we saw partial colocalization of FXR1P with FMRP, FXR2P, and argonaute 2 in P0 positive dendritic clusters (Figure S3). However, in most cases argonaute 2 clusters were located on the edge of the P0 clusters, whereas FXR1P occupied the majority of the P0 cluster. In contrast, PAK1 was ubiquitously expressed throughout the neuronal dendrites and axons of neurons, a pattern shown previously [48], and showed no specific colocalization with FXR1P (data not shown). These results demonstrate that a subset of FXR1P/P0 clusters also contain the RNA-binding proteins FXR2P, FMRP and argonaute 2.

### eGFP-FXR1P colocalizes with ribosomal subunits and mRNAs in clusters along the dendrite

**Figure 7:**
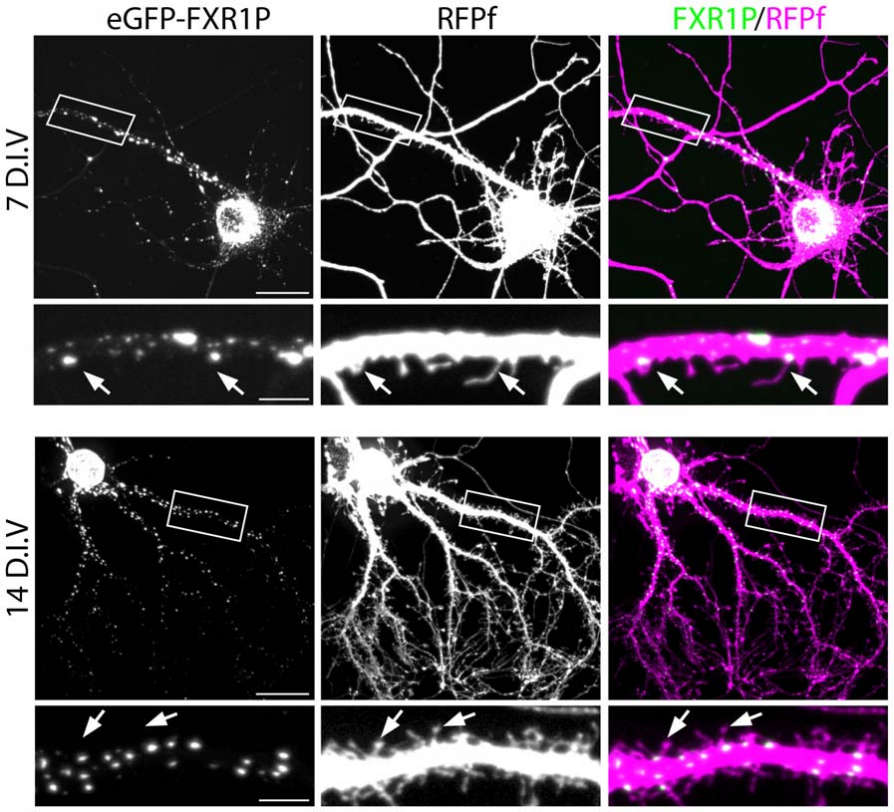
eGFP-FXR1P forms clusters along the dendrite and at spine-like protrusions in cultured neurons. We co-transfected hippocampal neurons grown for either 7 or 14 days *in vitro* with plasmids encoding membrane-targeted red fluorescent protein (RFPf) and eGFP-FXR1P. RFPf was used to visualize filopodia and spine-like protrusions. Here we show both low magnification and high magnification images of RFPf and eGFP-FXR1P at 7 and 14 days *in vitro.* We find that similar to endogenous FXR1P, overexpressed eGFP-FXR1P forms clusters of different sizes all along the dendritic shaft, with some of these clusters found close to filopodia and spine-like protrusions. Arrowheads point to filopodia and spines that are closely apposed by a bright eGFP-FXR1P cluster. Scale bar = 20 µm (low magnification) and 5 µm (high magnification). D.I.V = days *in vitro*.

We next tested whether a fluorescently tagged version of FXR1P would behave similarly to the endogenous protein when overexpressed in neurons at both 7 and 14 days *in vitro* (Figure 7). Similar to endogenous FXR1P, eGFP-FXR1P formed clusters of various sizes in the perinuclear region and dendrites. However, these clusters were often larger and more defined than the clusters seen with endogenous FXR1P staining. This was not due to aggregation of eGFP, since both untagged FXR1P and myc-tagged FXR1P showed similar cluster sizes when overexpressed in neurons (data not shown). Similar to endogenous FXR1P, eGFP-FXR1P clusters were found at the base of a subset of dendritic spine-like protrusions.

**Figure 8:**
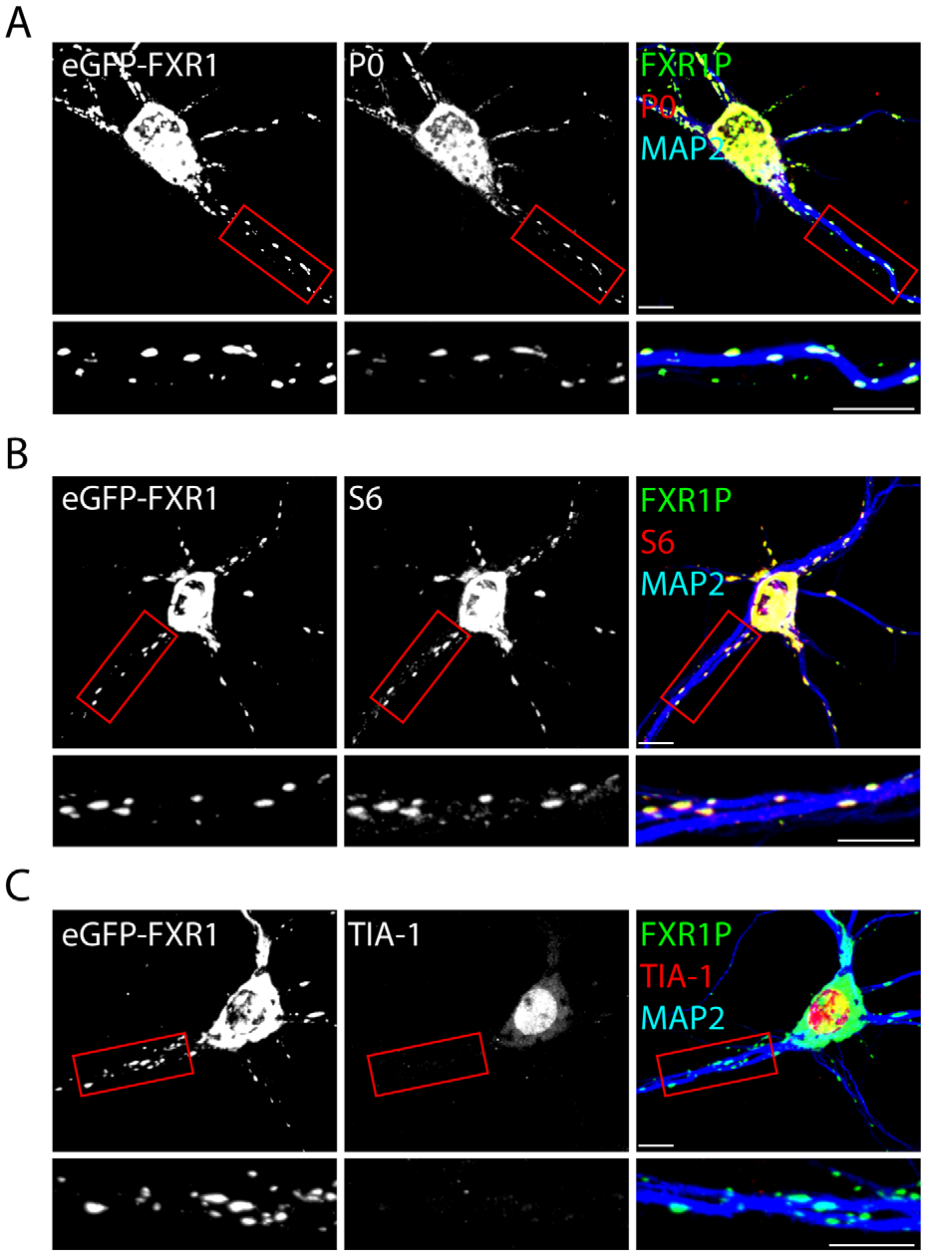
eGFP-FXR1P colocalizes with ribosomes. Dissociated hippocampal neurons were transfected with eGFP-FXR1P at 7 days *in vitro*. Cells were fixed after 24 hours and immunostained using an antibody against P0, a marker of the large ribosomal subunit (A), S6, a marker of the small ribosomal subunit (B), and TIA-1, an RNA-binding protein and marker of stress granules (C). In all cases, neurons were also immunostained with an antibody against MAP2 to delineate the proximal dendrites. We find that the majority of eGFP-FXR1P clusters contain strong signals for P0 and S6, but not TIA-1. The same results are seen at 14 days *in vitro* (data not shown). Results of the colocalization analyses are shown in Table 2. Scale bar = 10 µm.

We next asked whether these clusters contained ribosomal proteins. We immunostained neurons expressing eGFP-FXR1P with antibodies against the large and small ribosomal subunits (P0 and S6 respectively) and quantified the number of FXR1P clusters containing either P0 or S6 signal (Figure 8A, B and Table 2). The majority (∼70%) of FXR1P clusters contained correlated P0 or S6 signal. All other measures of colocalization also demonstrated high levels of colocalization (Table 2). Most surprisingly, we noted that the staining pattern of P0 and S6 changed to follow the cluster pattern of overexpressed FXR1P. Specifically, the clusters became larger, brighter and more defined upon FXR1P overexpression (compare P0 staining in Figure 8A with staining in Figure 4A). To rule out the fact that eGFP-FXR1P was forming a non-specific cluster of RNA binding proteins or a stress granule [49], [50], we repeated the analysis using staining against T cell immunoantigen-1 (TIA-1). TIA-1 is an RNA binding protein that normally resides in the nucleus and perinuclear region of non-neuronal cells and redistributes to stress granules when cells are stressed [50], [51]. We first verified that our antibody was capable of detecting TIA-1 positive stress granules in both heterologous cells and neurons challenged with puromycin or arsenite (Figure S4) [50]. We found that TIA-1 redistributed into cytoplasmic granules in stressed heterologous cells and neurons (Figure S4), demonstrating that our antibody does detect TIA-1 positive stress granules. In contrast, we found that overexpression of eGFP-FXR1P in neurons did not cause a redistribution of TIA-1 into cytoplasmic granules and TIA-1 was not colocalized with eGFP-FXR1P clusters (Figure 8C and Table 2). This indicates that overexpressed eGFP-FXR1P is not causing a general redistribution of RNA binding proteins or causing cellular stress.

**Table 2:** eGFP-FXR1P colocalizes with ribosomes and mRNA (Means, 95% confidence intervals).

	Pearson’s Coefficient	Mander’s 1^a^	Mander’s 2^b^	Intensity Correlation Quotient	% colocalization(SE)^c^
**FXR1P / P0** d	0.76 (0.71−0.82)	0.96 (0.92−0.99)	0.80 (0.77−0.89)	0.32 (0.30−0.34)	72.8 (3.5)
**FXR1P / S6** e	0.74 (0.65−0.83)	0.96 (0.92−0.99)	0.73 (0.65−0.80)	0.32 (0.28−0.34)	68.1 (3.9)
**FXR1P/mRNA** f	0.69 (0.61−0.80)	0.88 (0.84−0.92)	0.83 (0.75−0.92)	0.33 (0.30−0.35)	79.3 (N/A)
**FXR1P / TIA-1** g	−0.04 (−0.09−0.02)	0.27 (0.21−0.37)	0.27 (0.21−0.33)	0.17 (0.16−0.19)	23.5 (6.3)

a Overlap of FXR1P with label of interest.

b Overlap of label of interest with FXR1P.

c % colocalization (standard error) = # of FXR1P clusters with correlated signal from label of interest/total number of FXR1P clusters on dendritic segment.

d Number of independent cultures = 3; Number of cells = 22; Number of granules = 558.

e Number of independent cultures = 4; Number of cells = 28; Number of granules = 576.

f Number of independent cultures = 1; Number of cells = 12; Number of granules = 589.

g Number of independent cultures = 5; Number of cells = 51; Number of granules = 1367.

To determine whether the colocalization of FXR1P with ribosomes was unique to eGFP-FXR1P (isoform d), we repeated the P0 staining using untagged FXR1P, myc-tagged FXR1P, mCherry-FXR1P (isoform a), eGFP-FXR2P and eGFP-FMRP (isoform 1). We found that regardless of the tag, family member or isoform tested, these proteins formed clusters that contained high levels of P0 (data not shown) and hence likely reflect the true distribution of overexpressed Fragile X proteins.

In addition, we tested whether overexpressed Fragile X proteins colocalize in clusters with their endogenous counterparts (Figure S5). We found that eGFP-FXR1P partially colocalized with FXR2P in large clusters (Figure S5A). eGFP-FXR2P and eGFP-FMRP clusters both contained FXR1P (Figures S5B,C). We were unable to verify whether FXR1P clusters contain endogenous FMRP due to the slight cross-reactivity of antibody 1C3 with FXR1P [22]. These results demonstrate that over-expressed Fragile X proteins, similar to the endogenous proteins (Figure S3), retain their ability to colocalize with their endogenous counterparts in clusters.

**Figure 9:**
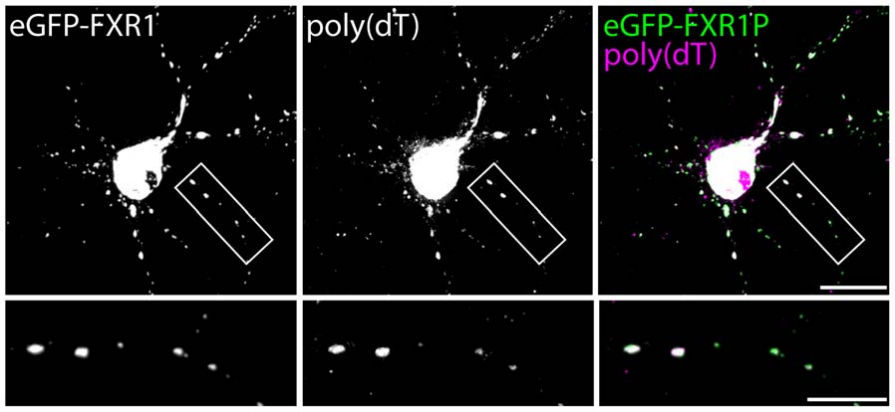
eGFP-FXR1P colocalizes with mRNAs. Dissociated hippocampal neurons were transfected with eGFP-FXR1P at 14 days *in vitro*. Cells were fixed after 24 hours and hybridized with a digoxigenin-labeled poly(dT) probe to detect polyadenylated mRNAs. *In situ* hybridization was followed by immunostaining for GFP and P0 (data not shown). We found that the majority of eGFP-FXR1P clusters contain mRNAs. Results of the colocalization analyses are shown in Table 2. Scale bar = 20 µm (low magnification) and 10 µm (high magnification).

Finally, we tested whether FXR1P clusters also contained mRNAs. We performed FISH with a poly (dT) probe on neurons transfected with eGFP-FXR1P. Similar to the ribosome staining, we found that the majority of FXR1P clusters (approximately 80%) contained mRNAs (Figure 9 and Table 2). Together, these results demonstrate that eGFP-FXR1P forms clusters containing ribosomes and mRNAs along the dendrite and at spine-like protrusions.

### eGFP-FXR1P clusters are found at the base of a subset of dendritic spines

**Figure 10:**
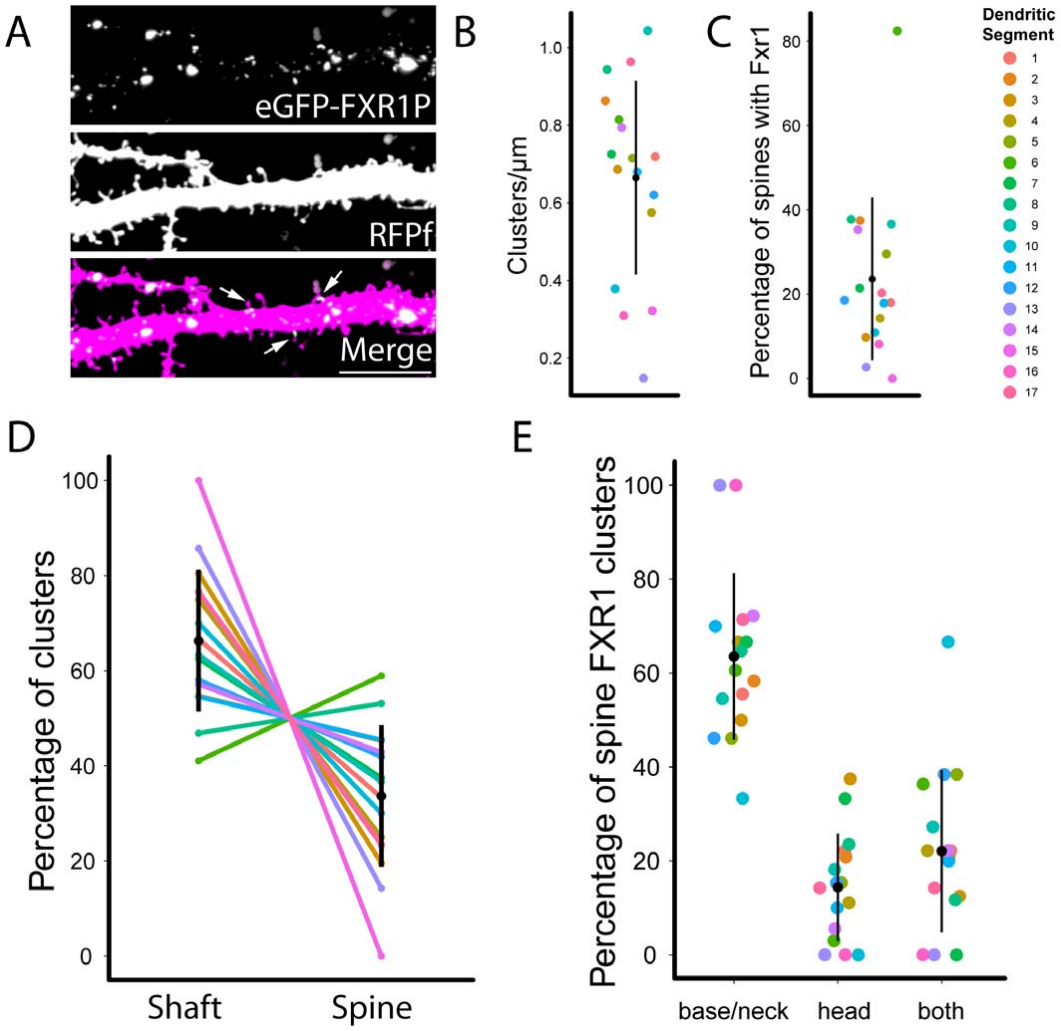
eGFP-FXR1P clusters are found at the base of a subset of dendritic spines. We transfected organotypic hippocampal slices at 7 days *in vitro* with plasmids encoding eGFP-FXR1P and membrane targeted red fluorescent protein (RFPf). The slices were fixed after 48 hours and CA1 apical dendrites were imaged using confocal microscopy. We quantified the subcellular localization of eGFP-FXR1P with respect to the dendrite and dendritic spines. **A.** A representative image of an apical dendrite of a CA1 cell. eGFP-FXR1P clusters are found along the dendrite and at a subset of spines. Arrows point to spines with a closely apposed eGFP-FXR1P cluster. **B.** We found that the density of eGFP-FXR1P clusters was variable and averaged 0.67±0.25 clusters/µm (mean±standard deviation(SD)). **C.** eGFP-FXR1P clusters were found at a subset of dendritic spines. On average, eGFP-FXR1P clusters were found at 23.6±19.34% of spines (mean ± SD). **D.** The majority of clusters were found in the dendritic shaft ( = 66.3%, spine = 33.7%). **E.** eGFP-FXR1P spine clusters are more likely found at the base and neck of the dendritic spine versus the spine head (base/neck = 63.5%, head = 14.4%). Each dendritic segment is color coded to allow comparison between the different measurements. The black dot and vertical bar represent mean ± standard deviation (SD). Data represent 17 dendrites imaged from 4 independent slice cultures.

Our previous experiments showed that both endogenous and overexpressed FXR1P are localized to the base of only a small number of spine-like extensions and are co-localized with protein synthesis machinery (see Figures 3C and Figure 7). To quantify the distribution of FXR1P clusters with respect to the dendrite and spines as well as to determine the proportion of spines containing FXR1P clusters, we transfected plasmids expressing eGFP-FXR1P and RFPf into organotypic hippocampal slices from mice. We chose to quantify the distribution of eGFP-FXR1P clusters instead of endogenous FXR1P clusters because we could focus our analysis on dendritic FXR1P clusters without influence from clusters found in neighboring cells. Furthermore, using exogenous eGFP-FXR1P, we could perform the analysis in organotypic slices, which provide a useful model system for studying dendritic spines on CA1 pyramidal neurons [39], [52]. Indeed, our previous work has shown that the majority of dendritic spine protrusions have associated presynaptic terminals and likely represent actual synapses [30]. Qualitatively, the distribution of eGFP-FXR1P clusters in slices was similar to that in dissociated hippocampal neurons (Figure 10A). Further analysis showed that eGFP-FXR1P cluster density was highly variable across the 17 dendritic segments analyzed (Figure 10B). The majority of clusters were found on the dendritic shaft and an average of 23.6% of spines contained at least one eGFP-FXR1P cluster (Figure 10C, D). Within this 23.6%, we found that FXR1P was more than twice as likely to be present at the base or neck of the spine than in the head of the spine (Figure 10E). Interestingly, the majority of eGFP-FXR1P clusters are immobile over time periods of 10 minutes to 1 hour in both young dissociated hippocampal neurons (Figure S6) and organotypic slice cultures (data not shown). These results indicate that FXR1P clusters are found at stable structures containing protein synthesis machinery and are located at the base of a subset of spines in hippocampal neurons.

## Discussion

The goal of the study was to determine whether FXR1P localizes with the translational machinery in the dendrite and at spines of mouse hippocampal neurons. Using biochemistry and confocal imaging with colocalization analysis, we demonstrate that FXR1P has enriched expression during hippocampal development and that the majority of FXR1P associates with polyribosomes and colocalizes with components of translational machinery including ribosomes and mRNAs in dendrites and at the base of a subset of dendritic spines. Our results support a role for FXR1P in local mRNA translation in neurons.

Local mRNA translation is regulated by RNA binding proteins which play a role at many different steps in the mRNA life cycle. In neurons, some mRNAs must be processed and trafficked out of the nucleus, repressed en-route to their destinations, and then stored safely until a signal is received, at which point they need to be rapidly translated and then stored again for future use or degraded [53]. Our study suggests that FXR1P may function in controlling mRNAs at multiple steps in neurons.

Firstly, we found that FXR1P is associated with polyribosomes in developing brain and localized with mRNAs in discrete clusters in the dendrites. In addition, we noted that the majority of these FXR1P clusters are immobile (Figure S6). The properties of these clusters are reminiscent of RNA granules – large aggregates of mRNAs, ribosomes and RNA binding proteins that are thought to store and traffic repressed mRNAs [54], [55], [56], [57]. In fact, the observation that overexpressing FXR1P increases the degree of co-localization with ribosomes and mRNAs, suggests that high levels of FXR1P can actively recruit ribosomes and mRNAs into RNA granules. This suggests that FXR1P could play a role in storing and protecting repressed mRNAs in neuronal RNA granules.

Secondly, FXR1P may function as a regulator of local mRNA translation. FXR1P is known to both repress and enhance the translation of target mRNAs in monocytes and macrophages depending on external cues [28], [29]. These findings raise the intriguing possibility that FXR1P may act as a switch for mRNA translation in response to external signals. This would be relevant for neurons, where synaptic activity leads to rapid local protein synthesis in dendrites [58], [59]. The stable localization of FXR1P with ribosomes at the base of dendritic spines is consistent with a role in controlling activity-dependent local protein synthesis [58], [59]. To address this possibility, future studies will be needed to determine if synaptic activity changes the localization or mobility of FXR1P clusters near spines and whether FXR1P can directly affect activity-dependent local mRNA translation. Indeed, this hypothesis fits well with results showing that synaptic activity can change the distribution of ribosomes, mRNAs and other RNA binding proteins in order to modulate local protein synthesis, remodel spines, and adjust synaptic strength [60], [61], [62].

Lastly, FXR1P could also play a role at the level of mRNA trafficking. In support of this, we found that the degree of co-localization was greatest with mRNAs versus the large ribosomal subunit. This was reflected by a minor fraction of small FXR1P clusters that did not contain discernible P0 staining (Figures 4D, E). These small, non-ribosome containing mRNA protein particles (mRNPs) may represent mRNAs trafficking from the nucleus to the dendrites and spines. Further experimentation is needed to test whether FXR1P is involved in trafficking mRNAs into the dendrites and spines, for example by reducing the level of FXR1P in neurons and tracking the fate of candidate target mRNAs.

A major unanswered question is the actual identity of mRNA targets of FXR1P in neurons. Previous studies have shown that FMRP and FXR1P both bind to kissing complex containing RNAs *in vitro*, suggesting that FMRP and FXR1P share some mRNA targets [63]. However, a more recent study using *in vivo* crosslinking-immunoprecipation to identify FMRP targets from mouse brain has questioned the view that FMRP binds to specific RNA structures since FMRP seems to be present along the entire length of target mRNAs [64]. Nevertheless, our results showing colocalization between FXR1P, FMRP and FXR2P in large dendritic clusters (Figures S3, S4) supports a model whereby FXR1P, FMRP and FXR2P cooperate to control the translation of certain neuronal mRNAs. If this is true, FXR1P, like FMRP, may regulate the translation of proteins important for building and maintaining the structure and function of the synapse.

To perform these diverse functions, FXR1P may coordinate with different protein partners including argonaute 2 and PAK1 in addition to FMRP and FXR2P [27], [29], [47]. Although FXR1P showed some partial colocalization with argonaute 2, we found that FXR1P and argonaute 2 showed mainly complementary expression patterns, with argonaute 2 being found at the edges of the P0 positive clusters (Figure S3). This localization pattern is consistent with reports of P-bodies (which contain argonaute 2) being closely located to, but non-overlapping with RNA transport particles or RNA granules [65]. We also did not observe selective colocalization between FXR1P and PAK1 in dendrites. It is possible that FXR1P may increase its interactions with argonaute 2 and PAK1 only under certain circumstances [29].

Currently, many aspects of FXR1P function in neurons remain unsolved, including its mechanism of action, its mRNA targets and its physiological importance. What might be the functional role of FXR1P at the synapse? Our results showing increased expression of FXR1P during early postnatal development of the mouse hippocampus suggests that FXR1P functions predominantly during synapse formation and synapse maturation. This is consistent with studies showing an important role for FXR1P in the early development of the eye, neural crest and muscle [66], [67]. Based upon our results, we propose that FXR1P is involved in local translational control of mRNAs in dendrites and may be involved in expressing proteins important for structural or physiological plasticity of dendritic spines. Further investigation is needed to determine how selective loss or overexpression of FXR1P in the brain affects neuronal and synaptic properties and whether FXR1P, like its homolog FMRP, is important for cognitive processes such as learning and memory formation.

## Supporting Information

Figure S1 **#ML13 is specific for FXR1P. A.** We transfected HEK 293T cells with plasmids encoding myc-tagged Fragile X proteins. We found that antibody #ML13 recognized FXR1P isoform d and did not cross-react with closely related family members FXR2 and FMRP. An antibody against myc confirmed that all proteins were successfully overexpressed. **B.** We immunostained cryostat sections prepared from a P18 td-tomato expressing mouse with #ML13 and secondary antibody only (Alexa Fluor goat anti-rabbit 647; Invitrogen) and imaged the hippocampus at 10X (left panel). Scale bar = 80 µm.
(TIF)

Figure S2 **poly (dA) control shows no staining.** Fluorescence *in situ* hybridization using a digoxigenin-labeled poly(dA) probe as an antisense control and immunostaining for FXR1P (#ML13). Brightness and contrast have been adjusted equally on the images to demonstrate the level of background staining from the poly (dA) probe. Scale bars = 10 µm.
(TIF)

Figure S3 **FXR1P partially colocalizes with FMRP, FXR2P and Argonaute 2 in clusters along the dendrite.** Immunostaining of dissociated hippocampal neurons at 14 days *in vitro* with anti-FXR1P (#ML13) and **A.** anti-FMRP (1C3), **B.** anti-FXR2P (A42) and **C.** anti-Ago2 antibodies demonstrates partial colocalization of FXR1P with these three known interacting proteins (P0 staining is also shown for comparison). Note that Ago2 also shows complementary staining with FXR1P, with Ago2 more likely to be found at the edges of the P0 clusters and FXR1P in the center. Graphs with labeled peaks demonstrating the covariance (or complementary staining in the case of Ago 2) in the fluorescence intensities along the dendritic segment are shown at the right. Scale bars = 10 µm.
(TIF)

Figure S4 **TIA-1 redistributes to stress granules. A.** COS-7 cells were treated with 20 µg/ml puromycin for 2 hours, followed by immunostaining for TIA-1. A small percentage of COS-7 cells display clearly visible TIA-1 positive cytoplasmic granules. Scale bar = 10 µm. **B.** Dissociated hippocampal neurons were treated with 0.5 mM arsenite for 30 minutes and immunostaining for TIA-1. Neurons showed the characteristic redistribution of TIA-1 into cytoplasmic granules. Scale bar = 10 µm.
(TIF)

Figure S5 **Fragile X Proteins colocalize with each other.** Dissociated hippocampal neurons were transfected with **A.** eGFP-FXR1P, **B.** eGFP-FXR2P and **C.** eGFP-FMRP at 7 days *in vitro*. Cells were fixed after 24 hours and immunostained using an antibody against **A.** FXR2P (A42), **B, C.** FXR1P (#ML13). **A.** Endogenous FXR2P partially colocalizes with eGFP-FXR1P in large clusters. **B, C.** Endogenous FXR1P colocalizes with eGFP-FMRP (B) and eGFP-FXR2P (C). Scale bars = 10 µm.
(TIF)

Figure S6 **FXR1P clusters are immobile. A.** Live hippocampal neuron transfected with RFPf and eGFP-FXR1P. **I, II, III.** Three examples of the FXR1P clusters imaged over time (images were taken every 8 seconds over 15 minutes). The majority of the FXR1P clusters were found to be immobile over this time-frame. Arrowheads denote immobile clusters while Arrows in I and II denote small clusters that were found to move over time. Scale bar = 10 µm.
(TIFF)
