# Supplementary figures and images for: Fragile X Related Protein 1 Clusters with Ribosomes and Messenger RNAs at a Subset of Dendritic Spines in the Mouse Hippocampus
Source: PLoS One. 2011 Oct 11;6(10):e26120. doi: 10.1371/journal.pone.0026120 (PMC3191184; doi:10.1371/journal.pone.0026120)

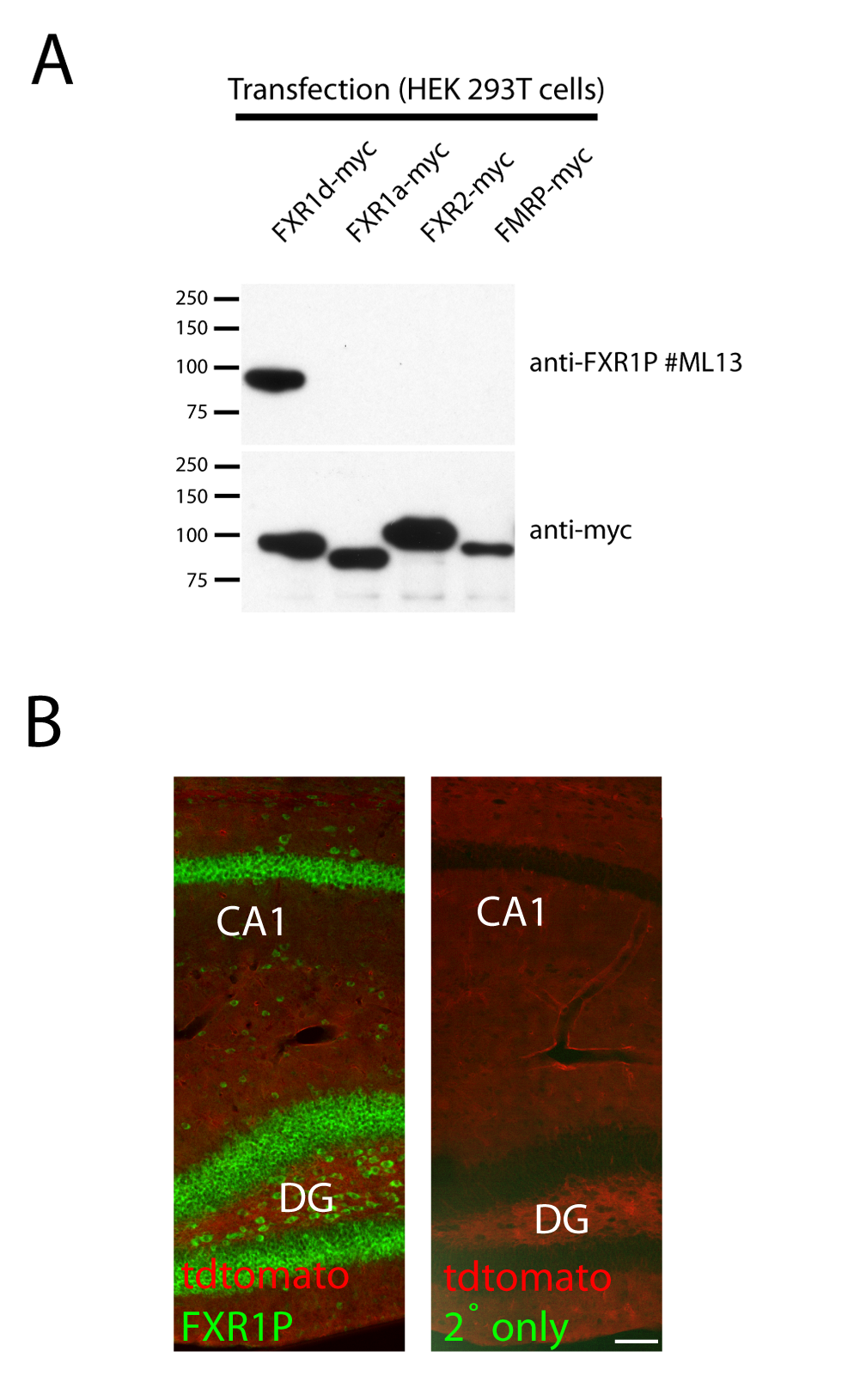

Supplement: Figure S1 — #ML13 is specific for FXR1P. A. We transfected HEK 293T cells with plasmids encoding myc-tagged Fragile X proteins. We found that antibody #ML13 recognized FXR1P isoform d and did not cross-react with closely related family members FXR2 and FMRP. An antibody against myc confirmed that all proteins were successfully overexpressed. B. We immunostained cryostat sections prepared from a P18 td-tomato expressing mouse with #ML13 and secondary antibody only (Alexa Fluor goat anti-rabbit 647; Invitrogen) and imaged the hippocampus at 10X (left panel). Scale bar = 80 µm. (TIF) [file pone.0026120.s001.tif]

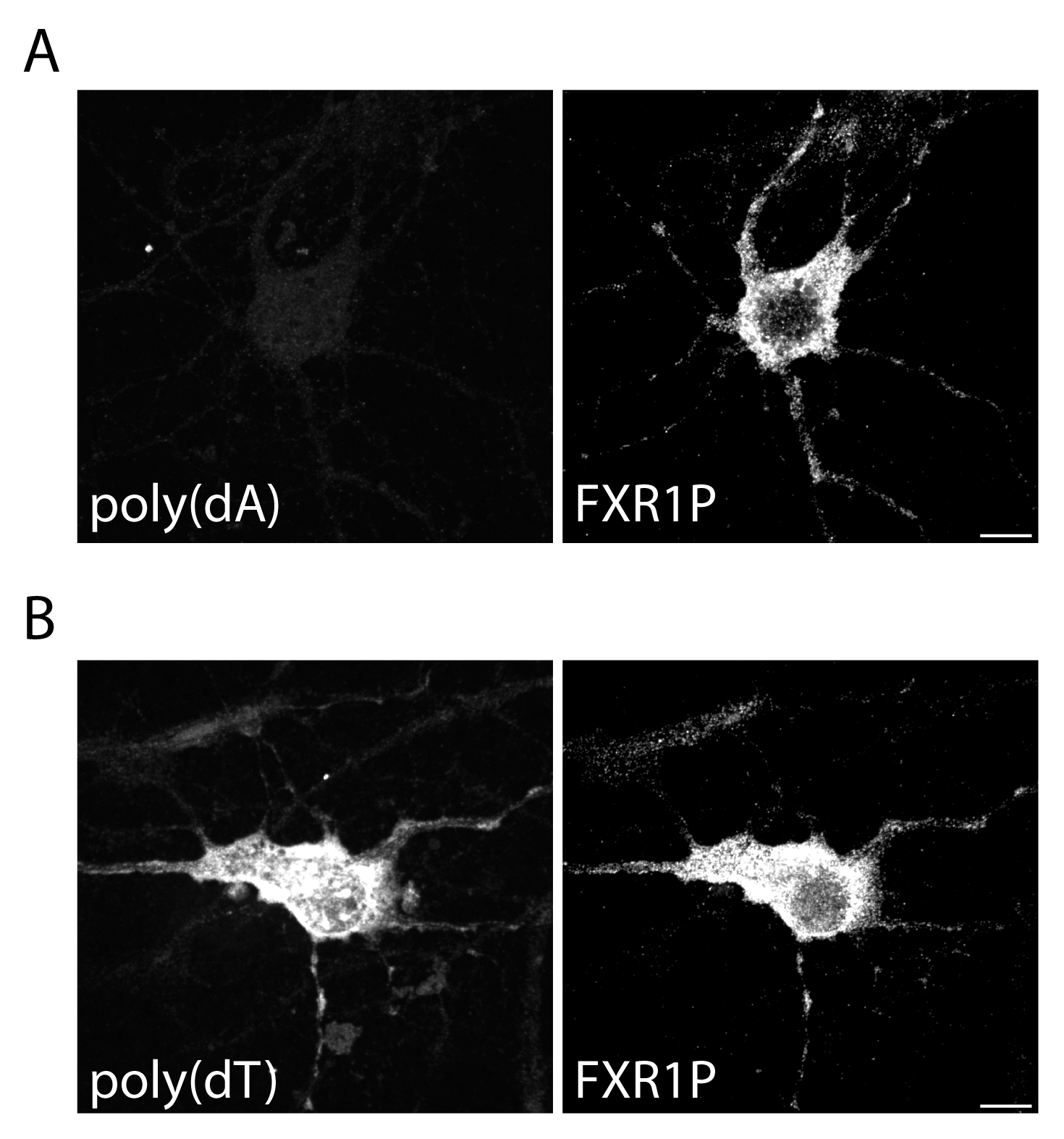

Supplement: Figure S2 — poly (dA) control shows no staining. Fluorescence in situ hybridization using a digoxigenin-labeled poly(dA) probe as an antisense control and immunostaining for FXR1P (#ML13). Brightness and contrast have been adjusted equally on the images to demonstrate the level of background staining from the poly (dA) probe. Scale bars = 10 µm. (TIF) [file pone.0026120.s002.tif]

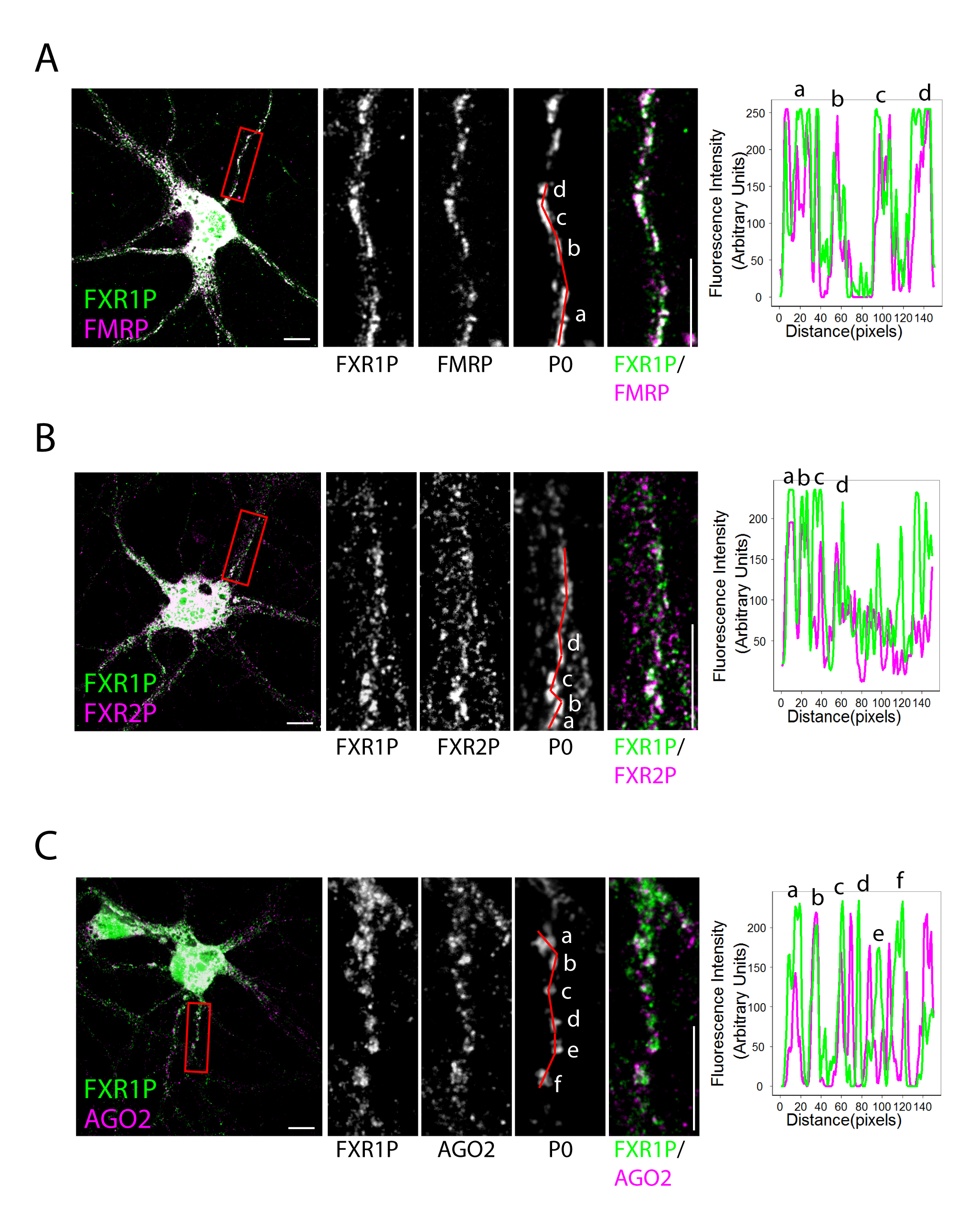

Supplement: Figure S3 — FXR1P partially colocalizes with FMRP, FXR2P and Argonaute 2 in clusters along the dendrite. Immunostaining of dissociated hippocampal neurons at 14 days in vitro with anti-FXR1P (#ML13) and A. anti-FMRP (1C3), B. anti-FXR2P (A42) and C. anti-Ago2 antibodies demonstrates partial colocalization of FXR1P with these three known interacting proteins (P0 staining is also shown for comparison). Note that Ago2 also shows complementary staining with FXR1P, with Ago2 more likely to be found at the edges of the P0 clusters and FXR1P in the center. Graphs with labeled peaks demonstrating the covariance (or complementary staining in the case of Ago 2) in the fluorescence intensities along the dendritic segment are shown at the right. Scale bars = 10 µm. (TIF) [file pone.0026120.s003.tif]

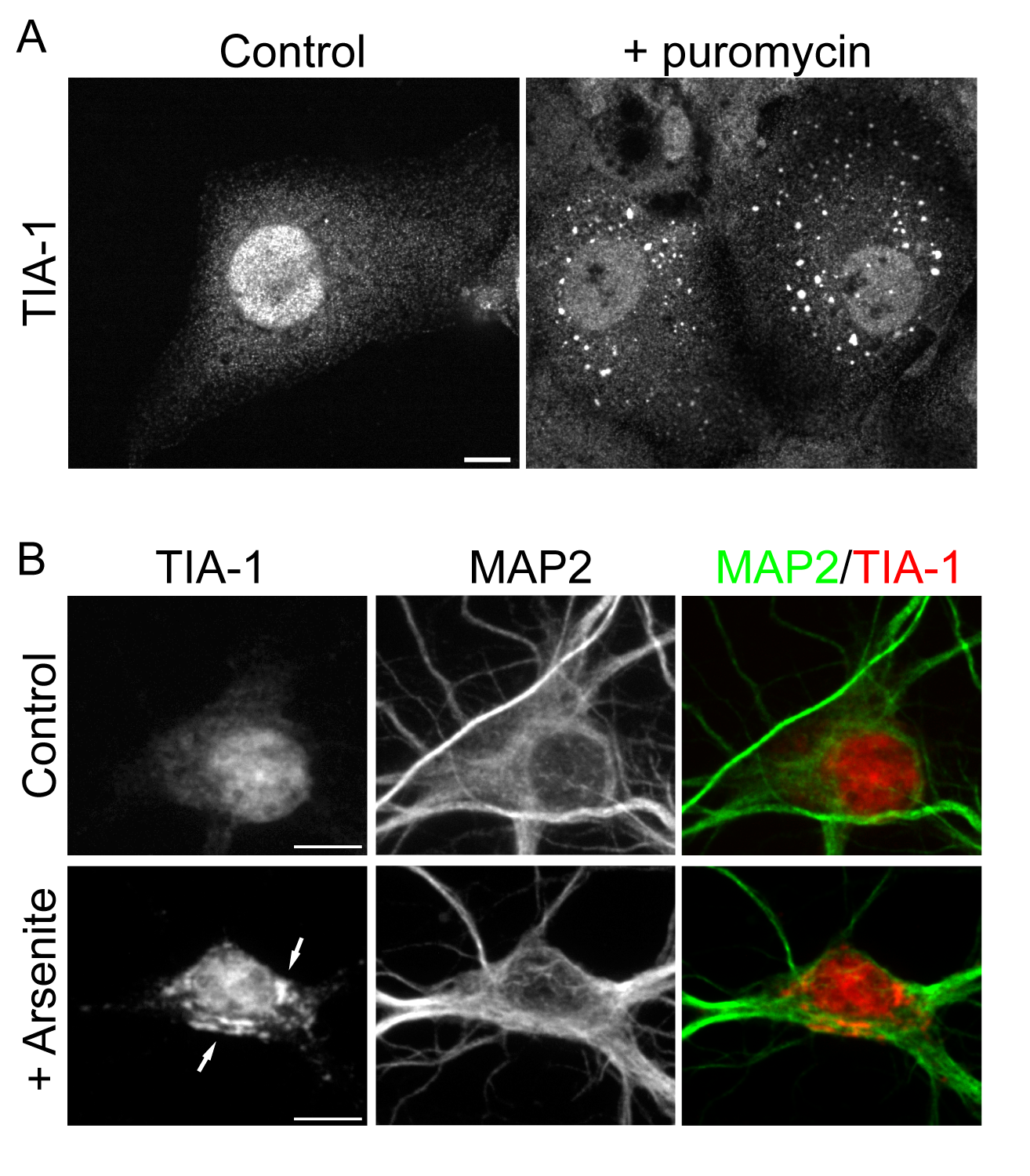

Supplement: Figure S4 — TIA-1 redistributes to stress granules. A. COS-7 cells were treated with 20 µg/ml puromycin for 2 hours, followed by immunostaining for TIA-1. A small percentage of COS-7 cells display clearly visible TIA-1 positive cytoplasmic granules. Scale bar = 10 µm. B. Dissociated hippocampal neurons were treated with 0.5 mM arsenite for 30 minutes and immunostaining for TIA-1. Neurons showed the characteristic redistribution of TIA-1 into cytoplasmic granules. Scale bar = 10 µm. (TIF) [file pone.0026120.s004.tif]

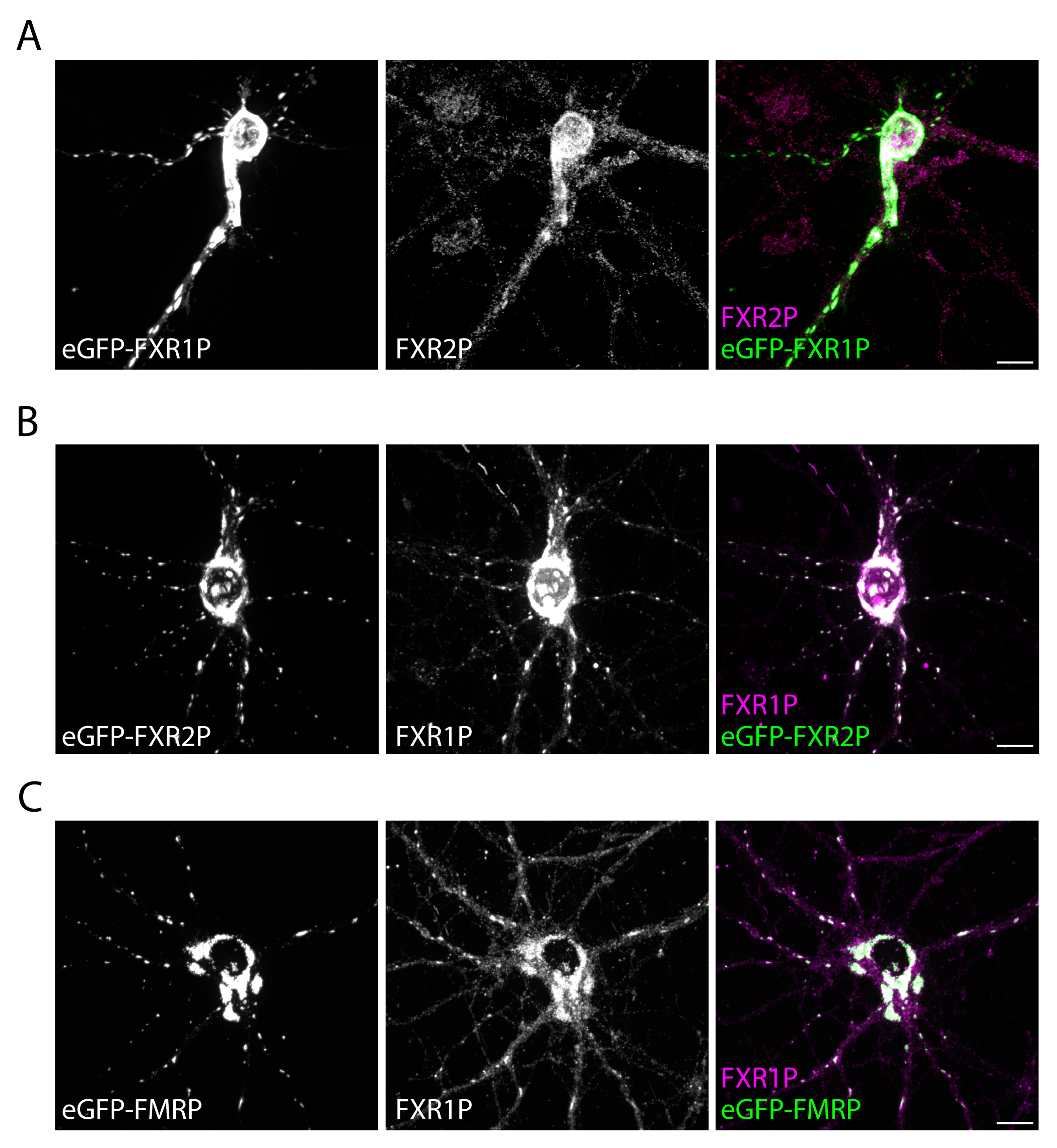

Supplement: Figure S5 — Fragile X Proteins colocalize with each other. Dissociated hippocampal neurons were transfected with A. eGFP-FXR1P, B. eGFP-FXR2P and C. eGFP-FMRP at 7 days in vitro. Cells were fixed after 24 hours and immunostained using an antibody against A. FXR2P (A42), B, C. FXR1P (#ML13). A. Endogenous FXR2P partially colocalizes with eGFP-FXR1P in large clusters. B, C. Endogenous FXR1P colocalizes with eGFP-FMRP (B) and eGFP-FXR2P (C). Scale bars = 10 µm. (TIF) [file pone.0026120.s005.tif]

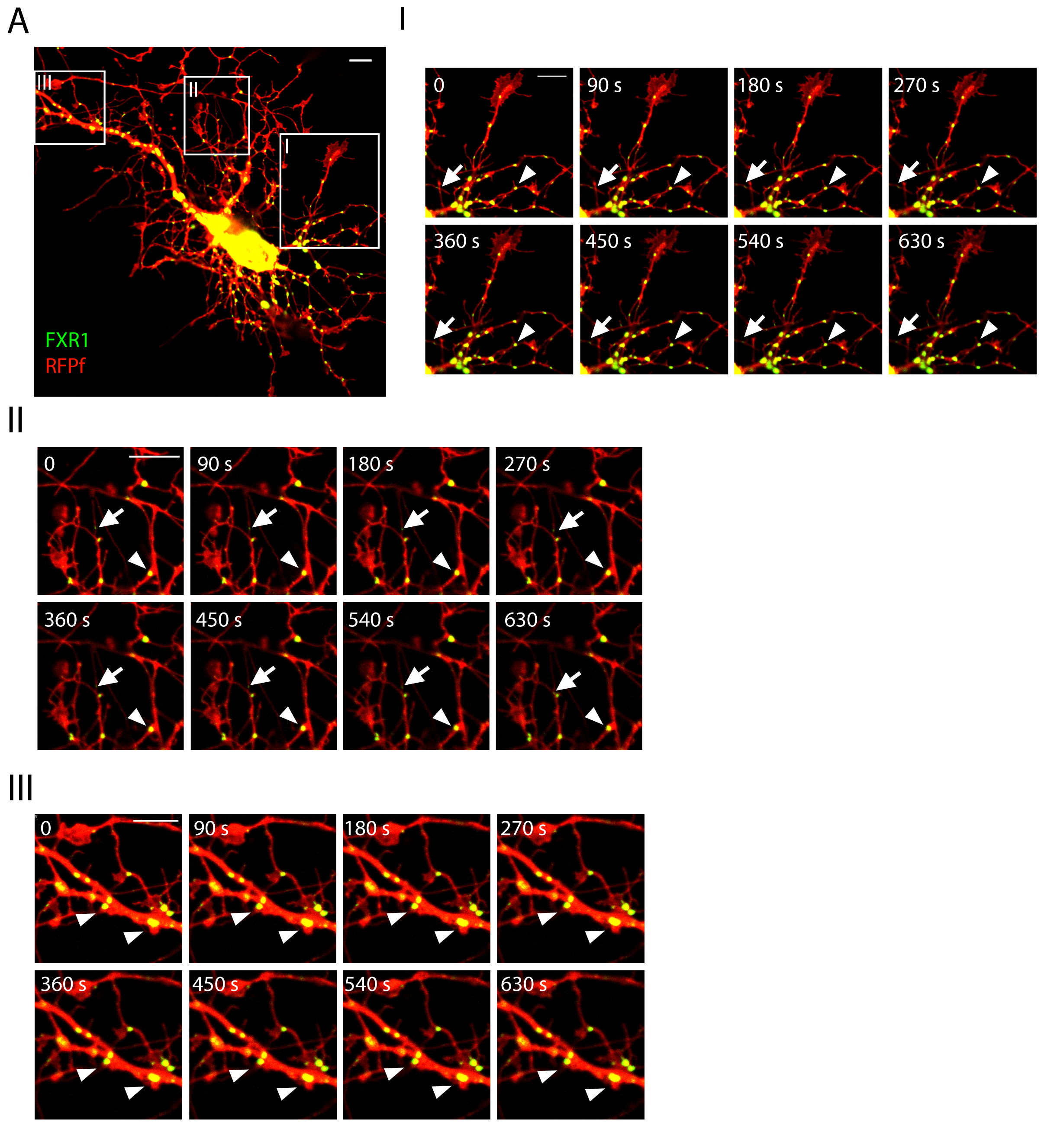

Supplement: Figure S6 — FXR1P clusters are immobile. A. Live hippocampal neuron transfected with RFPf and eGFP-FXR1P. I, II, III. Three examples of the FXR1P clusters imaged over time (images were taken every 8 seconds over 15 minutes). The majority of the FXR1P clusters were found to be immobile over this time-frame. Arrowheads denote immobile clusters while Arrows in I and II denote small clusters that were found to move over time. Scale bar = 10 µm. (TIFF) [file pone.0026120.s006.tiff]
